# SpectraClassifier 1.0: a user friendly, automated MRS-based classifier-development system

**DOI:** 10.1186/1471-2105-11-106

**Published:** 2010-02-24

**Authors:** Sandra Ortega-Martorell, Iván Olier, Margarida Julià-Sapé, Carles Arús

**Affiliations:** 1 Departament de Bioquímica i Biologia Molecular, Universitat Autònoma de Barcelona, UAB, Cerdanyola del Vallés (Barcelona), 08193, Spain; 2 Centro de Investigación Biomédica en Red en Bioingeniería, Biomateriales y Nanomedicina (CIBER-BBN), Cerdanyola del Vallès (Barcelona), Spain; 3 Institute of Neuroscience (INc), Autonomous University of Barcelona, UAB, Cerdanyola del Vallès (Barcelona), 08193, Spain

## Abstract

**Background:**

*SpectraClassifier *(SC) is a Java solution for designing and implementing Magnetic Resonance Spectroscopy (MRS)-based classifiers. The main goal of SC is to allow users with minimum background knowledge of multivariate statistics to perform a fully automated pattern recognition analysis. SC incorporates feature selection (greedy stepwise approach, either forward or backward), and feature extraction (PCA). Fisher Linear Discriminant Analysis is the method of choice for classification. Classifier evaluation is performed through various methods: display of the confusion matrix of the training and testing datasets; K-fold cross-validation, leave-one-out and bootstrapping as well as Receiver Operating Characteristic (ROC) curves.

**Results:**

SC is composed of the following modules: Classifier design, Data exploration, Data visualisation, Classifier evaluation, Reports, and Classifier history. It is able to read low resolution in-vivo MRS (single-voxel and multi-voxel) and high resolution tissue MRS (HRMAS), processed with existing tools (jMRUI, INTERPRET, 3DiCSI or TopSpin). In addition, to facilitate exchanging data between applications, a standard format capable of storing all the information needed for a dataset was developed. Each functionality of SC has been specifically validated with real data with the purpose of bug-testing and methods validation. Data from the INTERPRET project was used.

**Conclusions:**

SC is a user-friendly software designed to fulfil the needs of potential users in the MRS community. It accepts all kinds of pre-processed MRS data types and classifies them semi-automatically, allowing spectroscopists to concentrate on interpretation of results with the use of its visualisation tools.

## Background

### Introduction

Since the demonstration in 1989 that different brain tumour types displayed distinct spectral patterns [1], it became apparent that in order to determine whether in-vivo ^1^H-MRS had any clinical diagnostic value it was necessary first to gather a sufficiently large database of brain tumour ^1^H-MRS data and second, to perform statistical analysis of these multiple spectral features [2,3], which is frequently known as Pattern Recognition analysis (PR) or classification.

It was shown later on that it was possible to carry out a successful PR of the four most common brain tumour types, on a multicentre database of in-vivo single-voxel (SV) ^1^H-MRS data acquired at 1.5T [4]. The study was subsequently refined during the INTERPRET project [5], which successfully developed a PR-based decision-support system to assist radiologists in diagnosing and grading brain tumours using SV MRS data. However, the need for tools that allowed a rapid development of multiple classifiers for the already existing databases available [6] remained. This should also allow to rapidly test hypothesis that may surface during the lengthy process of data collection, especially in prospective studies [5]. This is especially relevant in the case of studies on human subjects [7], for instance with multi-voxel (MV) tumour data [8-10].

Moreover, the ever-increasing amount of biological data generated by metabolomics techniques also requires a tool allowing quick hypothesis testing on data that are difficult and expensive to gather [11-14]. In this sense, the PR analysis becomes just one stage in the iterative process of data-driven biological knowledge discovery.

However, ^1^H-MRS data are commonly analysed with either commercial (SPSS [15], SAS [16], SIMCA-P+ [17]), non commercial (R [18]) or home-made programs running over statistical packages of MATLAB [19], and usually require a certain degree of mathematical expertise for testing each individual hypothesis [20-22]. Some other packages for PR and classifier development (AMIX [23] and Pirouette [24]) are less complex tools, but commercial. Furthermore, Pirouette is platform-limited, because it is designed specifically for the Windows operating system.

Therefore, in order to facilitate the development of MRS-based classifiers, we developed *SpectraClassifier *(SC), a Java software solution to design and implement classifiers based on MRS data. The main goal of SC is to allow a user with minimum background knowledge of multivariate statistics to perform a fully automated PR analysis, from the feature extraction and/or selection stage to the evaluation of the developed classifier.

The purpose of this report is to describe SC, from the algorithms implemented to its main functionalities, with a focus on the different MRS data types it is able to work with. In addition, a standard format for exchanging either SV, MV or high-resolution MRS data for pattern recognition studies will also be described.

### Pattern recognition techniques

PR techniques aim to recognize and classify data (patterns) into different categories using the observed features. To do this, one of the possibilities is to base the development on a machine learning (ML) approach, in which a dataset is used to fit an adaptive model to solve the problem. ML provides the mathematical and computational mechanisms to infer knowledge in a formal model from specific data of a given domain [25].

**Figure 1:**
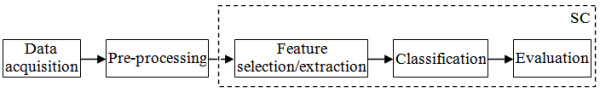
**Steps covered by SC in a pattern recognition system**. Most pattern recognition systems can be partitioned into these steps: data acquisition, which in our case obtains either the SV, MV or high resolution MRS data; pre-processing, which converts the raw data in the time domain into processed spectra in the frequency domain with the preferred pre-processing routines and protocols of choice; feature selection/extraction, to measure data vectors properties that are useful for classification; the classification, that uses these features to assign the data vector analysed to a category; and the evaluation, which assesses the model created. SC performs the last three steps (dotted box).

The life cycle of a PR problem based on ML is composed of two main phases: the training phase and the recognition phase. During the training phase, a set of signals from the problem domain is used to adapt a mathematical function to the output values, e.g. diagnosis, treatment, doses or risk. In this phase, the pre-processing and the features obtained from the signals are established, and the adaptive model is fitted, selected and evaluated in order to obtain the best generalization for solving new cases. Once the model is ready, it can be used for the recognition of new cases. Figure 1 shows a diagram of components of a typical pattern recognition system [26], and which of these components are covered by SC: feature selection and/or extraction (to decide which set of features can best determine class membership of a case), classification (to choose which is the classification model that best separates those classes) and evaluation (to find out how well this classification model will work with new data).

#### Selecting and/or extracting features

Several Feature Selection (FS) or Feature Extraction (FE) methods based on pattern recognition have been applied to the significant part of the spectra (MRS frequencies, in this case), looking for a subset of relevant peak heights of typical resonances (ppm, "part per million") or a reduced representation set of combinations of them. By removing most irrelevant and redundant information from the data, the valuable selected features help to improve the performance of learning models [27,28].

FE works by combining the existing data features into new ones that best describe the whole dataset according to a given criterion. It is, therefore, mostly a dimensionality reduction approach. FS, on the other hand, provides a selected subset of frequencies sufficient to classify tumour cases with reasonable accuracy.

SC implements two sequential FS methods based on a "hill climbing" search (Greedy Stepwise approach), either forward or backward, and evaluates the selected features with a CFS (Correlation-based Feature Subset) evaluator [29]. This Java class evaluates the worth of a subset of attributes by considering the individual predictive ability of each feature along with the degree of redundancy between them. Subsets of features that are highly correlated with the class while having low intercorrelation are preferred.

In addition to these FS methods, a FE method is also implemented: PCA (Principal Component Analysis). PCA performs a principal components analysis and transformation of the data, used in conjunction with a Ranker search (for ranking attributes by their individual evaluations). Dimensionality reduction is accomplished by choosing enough eigenvectors to account for a predefined percentage of the variance in the original data (we set it by default to cover a 95% of the variance, but this value can be modified by the user). Attribute noise can be filtered by transforming to the principal component space, eliminating some of the worst eigenvectors, and then transforming back into the original space.

#### Creating a classifier

The purpose of creating a classifier is to separate data vectors into one of two or more classes based on a set of features that better describe the data (features selected and/or extracted). In general, we assign a data vector to one of a number of classes based on observations made on the data. These classes are already known or predetermined.

At the moment, SC uses Fisher Linear Discriminant Analysis (Fisher LDA) as the technique of choice for distinguishing cases between two, three or four classes. Fisher LDA is a fundamental and widely used technique, that provides a reasonable way of reducing the dimensionality of the problem [26]. With the software, the user can assign each class to a different tumour type, or to a super-class generated by grouping tumour types [30].

As the original version of Fisher LDA does not assume any probability distribution to define the model, the limitation of Fisher LDA for estimating the probability of a case of belonging to a class, has been overcome by approximating the resulting projections through spherical Gaussian distributions, one for each class. The centre of each distribution has been assumed as the class mean estimated from data and the standard deviation common to all. Therefore, the probability of membership of every case to each class is estimated applying the Bayes' theorem over these distributions [26].

#### Evaluating models

An essential part of the life cycle of a classifier is its validation. To do that, there are evaluation methods to estimate how well the model will work with new but similar data in the future. SC implements some of the most commonly used methods for evaluating MRS-based classifiers. Those are briefly described below:

- Confusion matrix: each row of the matrix represents the members in a predicted class, while each column represents the actual value of members in the original class. It is a visualisation tool mainly used in supervised learning.

- Cross-validation: one round of cross-validation involves partitioning a dataset into complementary subsets, performing the training on one subset, and validating the model on the other subset. In K-fold cross-validation, the original dataset is partitioned into K subsamples. Of the K subsamples, a single subsample is retained as testing data for testing the model, and the remaining K-1 subsamples are used as training data. The cross-validation process is then repeated K times (the folds), with each of the K subsamples used exactly once as testing data. The K results from the folds can then be averaged to produce a single estimation of prediction accuracy [26]. In SC, the K value can be set by the user. It is typically used in scenarios where the goal is prediction, and it is desirable to estimate how accurately a predictive model will perform in practice.

- Leave-One-Out (LOO): is a special case of a K-fold cross-validation. It uses a single case from the original dataset as testing data, and the remaining cases as training data. This is repeated such that each case in the dataset is used once as testing data. This is the same as a K-fold cross-validation with K being equal to the number of cases in the original dataset.

- Bootstrapping: it is implemented by constructing a number N of bootstrap cases of the observed dataset (and of equal size to the training dataset), each of which is obtained by random sampling with replacement from the original dataset (there is nearly always duplication of individual cases in a bootstrap dataset). The N results from the bootstrap samples can then be averaged to produce a single estimation [26]. In SC, we set N equal to 1000 by default, but this value can be modified by the user. Bootstrapping could be better at estimating error rates in a linear discriminant problem, outperforming simple cross-validation [31].

- Receiver Operating Characteristic (ROC) curve: is a graphical plot of the sensitivity (True Positive Rate, TPR) vs. 1-specificity (False Positive Rate, FPR) for a binary classifier system as its discrimination threshold is varied [32].

## Implementation

SC is aimed at being an intuitive and user-friendly software tool. It has been built using the Java programming language (Java Platform, Standard Edition 6), ensuring the platform independence of SC. JFC/Swing classes were used to provide a Graphical User Interface (GUI) for the program. Java Runtime Environment 1.6 or later version is required to run the program.

In addition to the implementations of the methods described above, SC uses some open-source and well-known libraries, such as: Weka [33], a collection of machine learning algorithms, used in SC for selecting and extracting features; JavaStat [34], developed for performing basic statistics, used in SC for the implementation of the classification method (specifically the Discriminant Analysis class [35]); and KiNG (Kinemage, Next Generation) [36], which is an interactive system for three-dimensional vector graphics, and is used in SC to visualise the canonical variables or the components projection.

For exchanging data between applications, the development of a standard format capable of storing all the information needed for a dataset in a readable way was required. The selected language to create this format was XML (Extensible Markup Language) [37], the meta-markup language developed by the World Wide Web Consortium (W3C) which provides a general method of representing structured documents and data in the form of lexical trees.

## Results

### Main capabilities of SC

**Figure 2:**
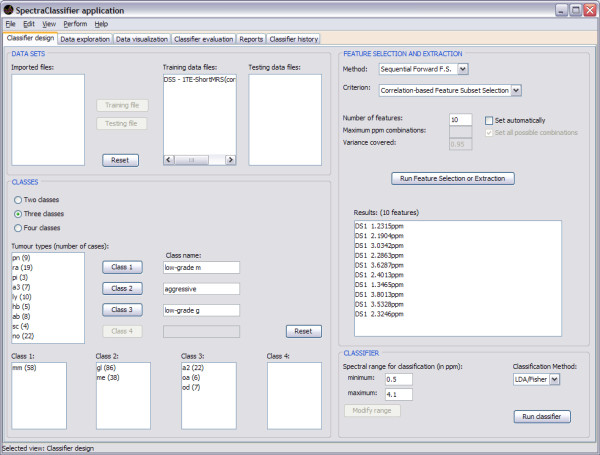
**Flow chart representing the construction and validation of a classifier using SC**. For developing a classifier using SC, the user can start by defining the training datasets, and then can follow this flow chart to develop a reliable and validated model.

SC is composed by the following modules or tabs: (TAB1) Classifier design, (TAB2) Data exploration, (TAB3) Data visualisation, (TAB4) Classifier evaluation, (TAB5) Reports, and (TAB6) Classifier history. Figure 2 is a flow chart that contains the main activities and transitions involved in the construction and validations of a classifier using SC.

In this section, the standard format definition for exchanging data and the main capabilities of SC will also be described. The development of a classifier will also be illustrated through computer screenshots. At the end of this section, some annotations about the validation with real data and computational consumption of the SC will be provided. For more detailed technical information about SC, please see the help and manual of the software in the Additional file 1.

### Standard format definition for data exchange

**Figure 3:**
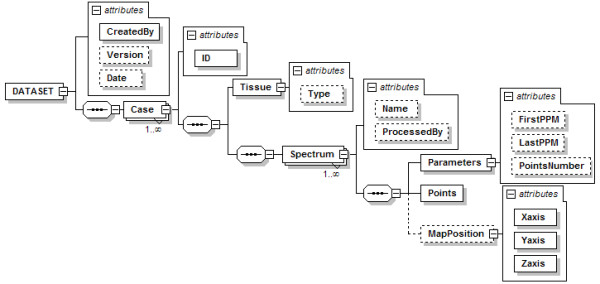
**Structure of the DATASET node**. The global node is *DATASET*, and is composed by one or more *Case *nodes. A *Case *node has an *ID *attribute for the identification of the case, and a sequence of nodes: first the *Tissue *node, with a *Type *attribute for the tumour type; and then a sequence of one or more *Spectrum *nodes. Every *Spectrum *node has three child nodes: *Parameters*, *Points*, and *MapPosition*. The *Points *node is used to store the spectral quantitative data, i.e. the intensity value of each point in the frequency domain, and the *MapPosition *node is used to store the x-y position of each spectrum in each MV grid. Dashed lines are used to indicate non-mandatory elements.

Figure 3 shows the structure of the standard format developed to describe MRS data.

### Classifier design

**Figure 4:**
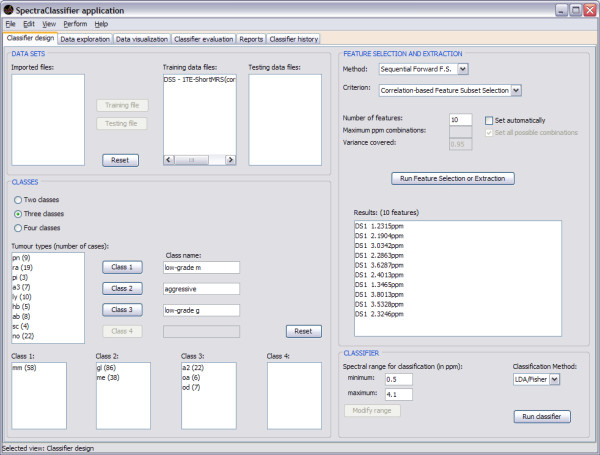
**Classifier design tab**. The training data are imported into the "DATA SETS" frame. The "Imported files" can be assigned to either "Training data files" or to "Testing data files" by clicking on the respective buttons. The "CLASSES" frame allows selecting and combining cases to be used for the classifier as training and to establish their name and composition. On "Class name", one can write down the name of the desired class. "Tumour types (number of cases)" displays the number of cases of each type in the training dataset, which can be assigned to the preferred class for classification. Several types already set in the "Training data files" can be merged into the same classification class, therefore allowing different combinations of training data types, for hypothesis testing. The "FEATURE SELECTION AND EXTRACTION" frame allows choosing the desired feature selection or extraction technique and the evaluation method. In this example the "Sequential Forward FS" and "Correlation-based Feature Subset Selection" have been chosen. Clicking on the "Run Feature Selection or Extraction" button below gives the resulting features. "DS1" means "Dataset one", since it is possible to concatenate two spectra from the same case obtained under different acquisition conditions and therefore the first one entered would be DS1. The "CLASSIFIER" frame allows the user to choose the spectral range (in ppm) which will be the desired region of interest for feature selection or extraction and for classification. The "Run classifier" button allows starting the classification with the selected "Classification method" (currently, Fisher LDA).

When SC is launched, Classifier design (Figure 4) is the first tab that can be seen. This tab allows the user to tune the desired input parameters for designing a classifier, such as the training datasets, the definition of classes and the selection or extraction of relevant features, which will be used as classifier inputs.

The development of a three-class classifier using SC is demonstrated throughout this report, using for this example a short TE SV training dataset of MR brain tumour data from INTERPRET [5,6,38]. In the example, "Class 1" is named *low-grade m *and contains 58 cases of the meningiomas (*mm*) type; "Class 2" is named *aggressive *and contains 86 cases of the glioblastoma multiforme (*gl*) type and 38 cases of the metastases (*me*) type; and "Class 3" is named *low-grade g *and contains 22 cases of the low grade astrocytomas (*a*2) type, 6 cases of the oligoastrocytomas (*oa*) type and 7 of the oligodendrogliomas (*od*) type.

#### Importing and exporting datasets

**Figure 5:**
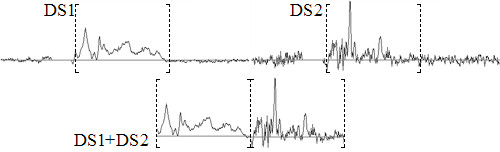
**Using two spectra by case**. When using two spectra by case (for instance when having two acquisitions at two different TEs) the new spectrum will be formed concatenating the range of interest (bracketed intervals) of both spectra.

There are two ways to import data, depending on whether the user wants to work with one spectrum per case or if he/she wants to make a combination of two spectra by case and merge them for analysis. When working with one spectrum per case, a matrix will be created with each row corresponding to the spectrum of the case and using only the selected range of interest of the spectrum. In cases of two spectra by case, a matrix will be created in a similar way as when there is one spectrum per case, but in each row, the first spectrum will be followed by the second one only in the range of interest that has been previously selected (Figure 5).

For importing dataset files, the preferable format is XML with the structure described before. It can be used for the three types of MRS data allowed by SC. Other formats can also be used to import dataset files, according to the type of MRS data:

1. In-vivo SV data, usually with a low number of points per spectrum (512-2048):

1.1. File with extension .txt or .art in the INTERPRET [5] canonical format, with 512 points in the [7.2; -2.8] ppm range, which only contains the information of one spectrum in one row. Similarly, files with extension .dat, exported with SPSS or similar, and composed by rows of 514 tokens, where the first row is columns labels (not used in SC), and the rest of rows correspond to cases (similar to the INTERPRET canonical format), having the following information each: identifier of the class, identifier of the case, and 512 points of the spectrum.

1.2. File with extension .txt, processed and exported using the Magnetic Resonance User Interface package (jMRUI) [39]. It is composed by a header and a four-column matrix of data. The header is partially used by SC, because it contains the number of points of the spectrum (*PointsInDataset*), and the information that allows inferring the spectral range (*SamplingInterval *and *TransmitterFrequency*). From the data matrix, only the third column (*fft (real)*) is read by SC.

2. In-vivo MV data, also with a low number of points per spectrum (512-2048), but with a large number of spectra per acquisition (n × n). SC treats each acquisition as one dataset:

2.1. File with extension .bsp, that corresponds to data pre-processed with 3D Interactive Chemical Shift Imaging v1.9.10 (3DiCSI) [40], and exporting the data in ASCII format [41]. It has the following structure: first row for the name of the set (not used in SC), line-break, *Number of voxels: *(a number), line-break, *Number of points per voxel: *(a number), line-break, *Voxel Index: *(with the information of the location of each voxel in a map, it is not used by SC), line-break, and then two columns with *Real *and *Imaginary *data.

3. High resolution data, usually with a large number of points per spectrum (16-32 K points).

3.1. File with extension .txt, for HRMAS. The original file having been processed with TopSpin [42] or similar and exported as text file. The number of points accepted is variable; the most commonly used are from 1600 to 3200, with a [4.5; 0.5] ppm range. Each file only contains the information of one spectrum in one column.

**Figure 6:**
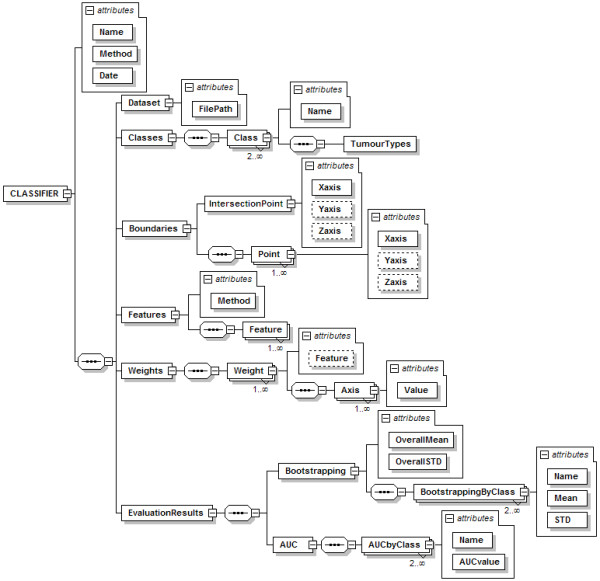
**Structure of the CLASSIFIER node**. The *CLASSIFIER *node has attributes for naming the classifier, indicating the classification method and the creation date; and it is composed by a sequence of six nodes: *Dataset*, *Classes*, *Boundaries*, *Features*, *Weights*, and *EvaluationResults*. The Dataset node has only the path to the dataset file. The *Classes *node contains a series of *Class *nodes for storing the tumour types involved in each class. The *Boundaries *node is for storing the points that form the boundaries between classes in the projection space: they are the intersection point (*IntersectionPoint *node) and the rest of points (the *Point *node sequence) used to draw a line from each of them to the intersection point. The *Features *node has the attribute *Method *for the name of the FS/FE method used, and the list of the resulting features. The *Weights *node contains the sequence of weights of the classifier, and the associated feature to each of them. The *EvaluationResults *node is for storing information related with the evaluation of the model, in this case, using bootstrapping (the *Bootstrapping *node) and the ROC curve (the *AUC *node). The *Bootstrapping *node has two attributes for the overall mean and standard deviation, and a list of nodes with the bootstrapping results per class. The AUC node contains a sequence of nodes with the *AUC *results by class. Dashed lines are used to indicate non-mandatory elements.

The pre-processing tasks needed for imported files are out of the scope of SC, therefore they have to be carried out before using SC, including adjustments of sweep width and number of points if spectra from different manufacturers are to be used. On the other hand, all imported datasets, training and testing sets, regardless of its original format, can be exported in the XML file format described before. Figure 6 shows the format in which the information of the classifier will be exported.

### Data exploration

**Figure 7:**
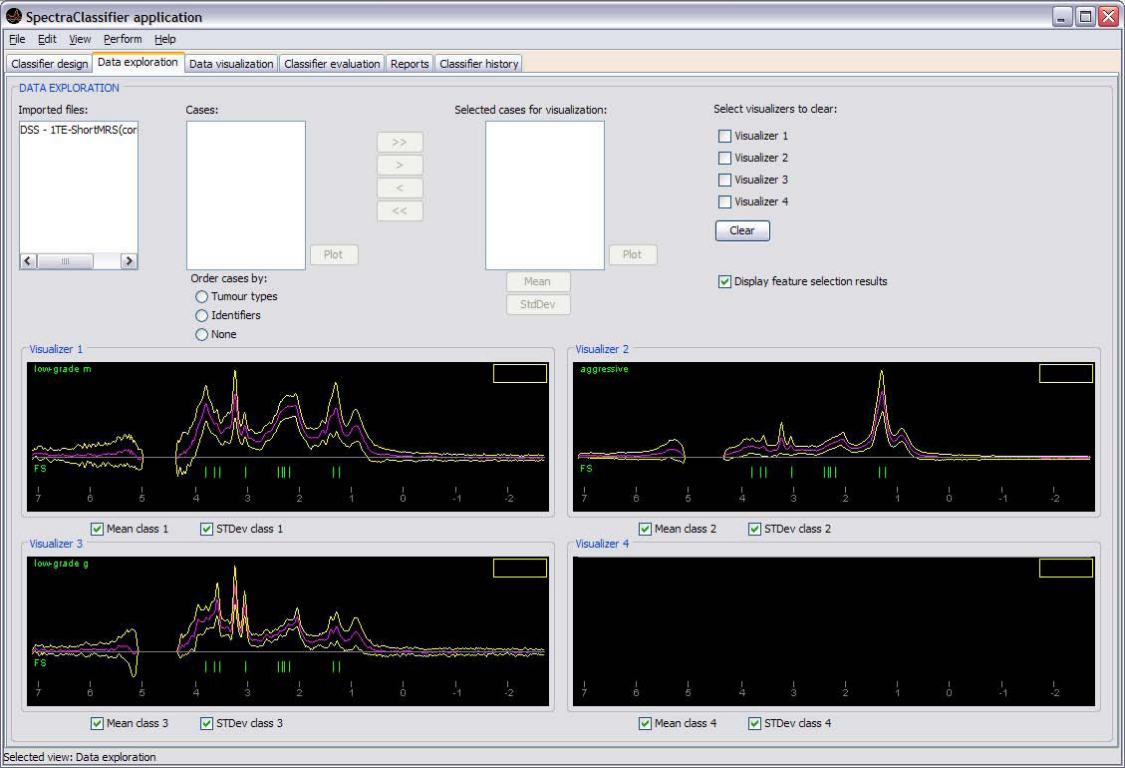
**Data exploration tab**. Continuing the example introduced in Figure 4, three of the four visualisers are showing the feature selection results: the mean (pink spectrum), the standard deviation range (yellow line) and the selected features (green vertical lines). Each visualiser displays the information of one class. The name of the class is written on the top left of the visualiser.

Data exploration is the second tab of the application. It can be used to plot the spectrum of individual cases, to plot the mean and the standard deviation of a set of cases, and to display the features obtained in the FS process (Figure 7).

### Data visualisation

**Figure 8:**
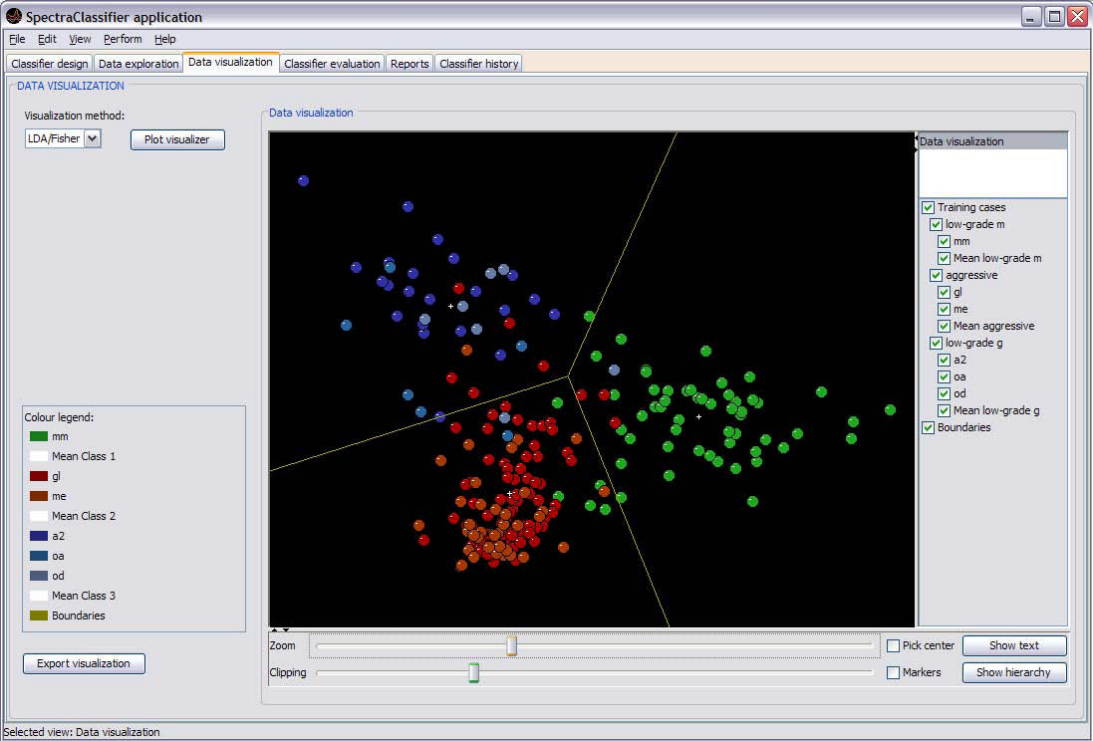
**Data visualisation tab**. Continuing the example of Figure 4, the projection space of the Fisher LDA classifier can be seen: *low-grade m *(*mm*, in green), *aggressive *(*gl*+*me*, in shades of red) and *low-grade g *(*a2*+*oa*+*od*, in shades of blue). This visualisation is a two-dimensional representation of the corresponding point in the space of each case, taking advantage of this visualisation by rotating it and twisting it around (using the mouse and the controls at the bottom of the visualisation panel), turning on or off parts of the display (using the check buttons components in the right of the visualiser), and identifying cases by selecting them with the mouse. As this example is a three-class classifier, a 2D display with the boundaries of the classes (yellow lines) is displayed.

Data visualisation is the third tab of the application (Figure 8). This tab can be used to visualise the position in the projection space of each case from the training and test sets after PCA (the projection of two or three components chosen by the user) or Fisher LDA (the projection of the canonical variables derived from the discriminant analysis) in two or three dimensions.

The implementation of this visualisation uses the KiNG library, which is called from SC to load a preformatted kinemage made automatically with the information of the data visualisation. In cases of one or two dimensions (when creating two-class or three-class classifiers), the boundaries of the classes will be calculated and displayed (Figure 8).

### Classifier evaluation and Reports

**Figure 9:**
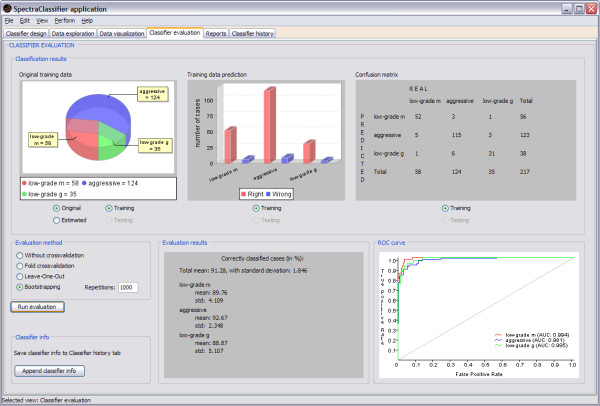
**Classifier evaluation tab**. In this example (started in Figure 4), the top left graph is a pie plot that can be used to check the global information of the number of cases that originally belong to each class, and the number of cases that the classifier predicted to belong to each class. The top centre graph is a bar plot used for checking the numerical relationship between rightly (the red ones) and wrongly (the blue ones) predicted cases per class. The top right panel is a confusion matrix, useful for checking predicted cases in each class. For example: the *low-grade m *class actually contains 58 cases, but the classifier predicts 52 of them as *low-grade m*, the other 6 are predicted to be aggressive (5) and *low-grade g *(1). The confusion matrix can also be generated for an independent test set, improving the capabilities of the evaluation. The bottom centre panel shows the bootstrapping results for N = 1000 (a total mean accuracy of 91.28%, with a standard deviation of 1.846%). The bottom right graph is the ROC curve (in the case of a classifier with more than two classes, like the one on this example, data are analysed by dichotomisation [32]), showing the plot of a ROC curve and the AUC value per class.

The Classifier evaluation tab in SC (Figure 9) has been developed with the purpose of identifying how well the classifier developed by the user performs and how robust it will be. Current evaluation methods implemented in SpectraClassifier just take into account the classifier, not involving other stages such as the feature selection or extraction (these stages rely on their own evaluation criteria, such as [29]). This approach can be slightly optimistic, so it is recommended to perform several tests with different numbers of features or to use an independent test set before deciding on a final classifier.

**Figure 10:**
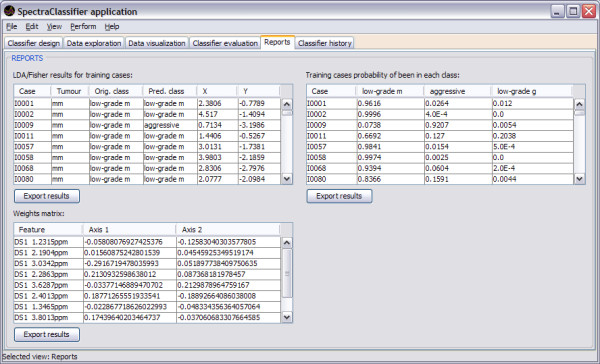
**Reports tab**. In this example three reports are shown. On the top left of this tab the Fisher LDA results for training cases are shown: each row of the table corresponds to one case, showing its identifier, the tumour types, the actual original class, the predicted class (obtained by the Fisher LDA method), and the corresponding X and Y coordinates for the representation in a projection space. On the top right the Fisher LDA probabilities results for training and testing cases are shown: each row of the table corresponds to one case, showing its identifier and the probabilities of belonging to each previously defined class (*low-grade m*, *aggressive*, *low-grade g*). In the bottom left there is the weights matrix report, showing the matrix of weights of the classifier, each of them associated to the corresponding spectral data vector feature (expressed in ppm).

The application also generates reports (in the form of tables) with the results obtained after creating a classifier, for training and testing data, and allows the user to export them as text files (Figure 10).

The Classifier history tab can store the main description of the classifiers chosen by the user. It can be used to compare these classifiers, checking variations of results obtained when developing classifiers with different parameters.

### Validation with real data

As it can be seen in the figures throughout the text, each functionality of SC has been specifically validated with real data with the purpose of bug-testing and methods validation. Data from the INTERPRET project [5] at short (see figures 4, 7, 8, 9 and 10) and long TE (results not shown) were used. Table 1 and 2 compile representative results of experiments conducted to validate the correct implementation and performance of the software. What was seen was that the results obtained with SC compare well with previous non-automated analyses of the same dataset [5,20-22,43,44]. Sample brain tumour data from real patients are distributed with SC for testing purposes.

**Table 1:** Comparing results for the validation of SC, using PCA prior to LDA.

	**Long TE **- [**20**]	Long TE - SC	**Short TE **- [**21**]	Short TE - SC
**Classes**	**AUC ± SE**	**AUC ± SE**	**AUC ± SE**	**AUC ± SE**

1 vs. 2	0.953 ± 0.031 _(8)_	0.977 ± 0.016 _(8)_	0.956 ± 0.028 _(4)_	0.923 ± 0.028 _(4)_

1 vs. 3	0.593 ± 0.104 _(6)_	0.757 ± 0.054 _(6)_	0.591 ± 0.097 _(4)_	0.688 ± 0.056 _(4)_

1 vs. 4	0.918 ± 0.063 _(7)_	0.941 ± 0.025 _(7)_	0.966 ± 0.029 _(3)_	0.962 ± 0.019 _(3)_

2 vs. 3	0.961 ± 0.038 _(5)_	0.970 ± 0.028 _(5)_	0.954 ± 0.044 _(4)_	0.972 ± 0.021 _(4)_

2 vs. 4	0.931 ± 0.073 _(10)_	0.999 ± 0.003 _(10)_	0.997 ± 0.009 _(11)_	1.000 ± 0.000 _(11)_

3 vs. 4	0.961 ± 0.053 _(4)_	0.995 ± 0.010 _(4)_	0.986 ± 0.025 _(2)_	0.979 ± 0.025 _(2)_

In this example, multiple binary classifiers were developed for long and short TE of SV MRS data from INTERPRET [5]. In [20,21] the PCA covered the 75% of the variance of the dataset. For SC, a variance of 80% has been covered. As performance measure, the AUC and its standard error (SE) were used. The number between brackets refers to the number of principal components used. The classes are: 1) glioblastomas, 2) meningiomas, 3) metastases and 4) astrocytomas grade II.

**Table 2:** Comparing results for the validation of SC, using FS prior to LDA.

	**Accuracy ± standard deviation in **[**44**]	Accuracy ± standard deviation in SC
Short TE	88.82 ± 4.51	90.73 ± 1.97

Long TE	82.50 ± 5.31	85.12 ± 2.51

Long + Short TE	88.71 ± 4.54	90.31 ± 2.16

In this example, a classifier for low grade meningioma, aggressive (glioblastomas multiforme + metastasis) and low grade glioma was developed for short, long and the combination by concatenation [44] of long + short TE of SV MRS data from INTERPRET [5]. In [44], k-Random sampling train-test (kRSTT) with stratified test sets with 150 repetitions was the evaluation procedure used. In SC, a bootstrapping method with 1000 repetitions was the one used. Although both methods used to evaluate the classifiers are not exactly the same, both are equivalent sampling methods, therefore their results can be compared. As performance measure, the accuracy and the standard deviation were used.

### Computational consumption

The computing time needed to develop a classifier depends on the dataset size. For example, in a 3 GHz CPU and 2 GB RAM personal computer, the typical performance values for INTERPRET 512-point files (188 points in the range of interest) in a classification problem with 217 cases and three classes are 4 seconds for feature selection with the sequential forward method and 3 seconds for Fisher LDA. For the same problem with concatenated spectra (i.e. 1024 points, 376 points in the range of interest, and 195 cases), times are 50 and 3 seconds, respectively. For HRMAS spectra of 1639 points (1310 points in the range of interest), feature selection takes 30 min with the same conditions. The computing time should be quite reduced using a high performance server.

## Discussion and conclusions

*SpectraClassifier *is a user-friendly software for performing PR of MRS data, which has been designed to fulfil the needs of potential users in the MRS community. It works with all types of MRS, i.e. SV, MV and high-resolution data (HRMAS). In addition, it also supports two concatenated spectra of the same resolution and number of points, since it had been previously shown that combination of data from two different TEs can provide useful additional information for classification [44,45].

SC allows easy data exploration, with four different spectra visualisers through which individual cases, class mean, standard deviation, as well as the selected classification features in each experiment can be explored. Classification results are shown both visually and numerically. The data visualisation tab allows feedback on classification errors through potential outlier analysis by using the four spectra visualisers.

The software is limited in two aspects: first, only very basic PR techniques have been implemented yet and second, at the moment its data reading capabilities span a few formats (i.e. data files that can be read by jMRUI).

With respect to the first limitation, it has been shown [22] for in-vivo SV 1H-MRS data, in a multicentre multiproject evaluation of classification methods for brain tumours that in fact most methods give comparable results. This has been as well shown in other PR challenges [20,21]. For this reason, we consider that the low number of methods implemented should not be considered as a drawback of SC.

Other widely used softwares [12-14] have also this limitation, such as SIMCA-P+, AMIX, and Pirouette, offering methods such as PCA, Partial Least Square (PLS), Soft Independent Modelling of Class Analogy (SIMCA), Principal Component Regression (PCR), and Classical Least Square (CLS).

With respect to the second limitation, i.e. format reading, in fact the lack of a common exchange format affects all areas of work of the MRS community, especially for clinical scanners. Although a standard DICOM had been defined [46], its implementation in output formats from in-vivo human scanners at 1.5 T (the most common in the clinic) is still far from being general. For this reason, we decided to leave the pre-processing step to the user (Figure 1) in order to minimise the number of different formats that have to be understood by SC. The program has been made compatible with the INTERPRET DSS software for in-vivo MRS data, which is accessible at no cost, upon signature of a disclaimer form [47]. Since INTERPRET developed a canonical format for in-vivo MRS data at 1.5T, it would therefore be possible for users with their own databases of 1.5T data to process data from different manufacturers and sweep widths and number of points with the DSS itself [48], and to export those into SC. At the same time, it is also possible to enter jMRUI-processed data into SC. jMRUI is able to read most existing clinical scanner formats. The future version of jMRUI (v 4.0), which will be able to accept plug-ins, should allow jMRUI and SC to connect with one another, adding the pre-processing step to the pipeline for MRS data analysis through the developed XML format. In this way, SC relies on existing processing software for format conversion and pre-processing and concentrates on the PR process.

Future SC developments include testing the software performance with other PR problems and with different data types (MV and HRMAS).

In conclusion, SC is a software that accepts all kinds of pre-processed MRS data types and classifies them semi-automatically, allowing spectroscopists to concentrate on interpretation of results with the use of its visualisation tools. The classifiers created can be exported as XML files for their easy implementation into decision-support systems (DSS), such as the INTERPRET DSS [5,47].

## Availability and requirements

Project name: *SpectraClassifier *(SC)

Project home page: http://gabrmn.uab.es/sc

Operating system(s): Platform independent

Programming language: Java

Other requirements: Java virtual machine 1.6.0 or higher

License: Available free of charge

Any restriction to use by non-academics: Subject to the signature of a disclaimer and user agreement text available at the project homepage.

## Abbreviations

AUC: Area Under the Curve; CFS: Correlation-based Feature Subset; CLS: Classical Least Square; DICOM: Digital Imaging and Communication in Medicine; DSS: Decision Support System; FE: Feature Extraction; FPR: False Positive Rate; FS: Feature Selection; JFC: Java Foundation Classes; LDA: Linear Discriminant Analysis; LOO: Leave-One-Out; ML: Machine Learning; MRS: Magnetic Resonance Spectroscopy; MV: Multi-Voxel; PCA: Principal Component Analysis; PCR: Principal Component Regression; PLS: Partial Least Square; PR: Pattern Recognition; ROC: Receiver Operating Characteristic; SC: SpectraClassifier; SIMCA: Soft Independent Modelling of Class Analogy; SV: Single-Voxel; TE: Echo Time; TPR: True Positive Rate; XML: Extensible Markup Language.

## Authors' contributions

SOM, IO and MJS participated in the design of the application. SOM implemented the software. IO contributed with the know-how in pattern recognition techniques. MJS and CA carried out major test of the software, and contributed with data interpretation and software validation. CA coordinated the work. All authors helped to draft the manuscript and approved the final version of it.

## Supplementary Material

Additional file 1 **Help and Manual of SpectraClassifier 1.0**. The "Help and Manual of SpectraClassifier 1.0" provides more detailed technical information about the software. Click here for file
