# Supplementary material for: SpectraClassifier 1.0: a user friendly, automated MRS-based classifier-development system
Source: BMC Bioinformatics. 2010 Feb 24;11:106. doi: 10.1186/1471-2105-11-106 (PMC2846905; doi:10.1186/1471-2105-11-106)
Supplement: Additional file 1 — Help and Manual of SpectraClassifier 1.0. The "Help and Manual of SpectraClassifier 1.0" provides more detailed technical information about the software. [file 1471-2105-11-106-S1.PDF]

SpectraClassifier 1.0 Help and Manual

v1.0 September 8, 2009

© 2009 GABRMN - Universitat Autònoma de Barcelona

Grupo de Investigación en Aplicaciones Biomédicas de la Resonancia Magnética Nuclear
Universitat Autònoma de Barcelona

SpectraClassifier 1.0 Help and Manual

by © 2009 GABRMN - Universitat Autònoma de Barcelona

SpectraClassifier is a Java solution for designing and implementing MRS-based classifiers. The main goal of SC is to allow users with minimum background knowledge of multivariate statistics to perform a fully automated pattern recognition analysis.

SpectraClassifier incorporates feature selection (greedy stepwise approach, either forward or backward), and feature extraction (PCA). Fisher Linear Discriminant Analysis is the method of choice for classification. Classifier evaluation is performed through various methods: display of the confusion matrix of the training and testing datasets; K-fold crossvalidation, leave-one-out and bootstrapping as well as ROC curves.

SpectraClassifier is composed of the following modules: Classifier design, Data exploration, Data visualisation, Classifier evaluation, Reports, and Classifier history. It is able to read low resolution (SV and MV) and high resolution MRS (HRMAS) processed with existing tools (jMRUI, INTERPRET, 3DiCSI or TopSpin). In addition, to facilitate exchanging data between applications, a standard format capable of storing all the information needed for a dataset was developed.

SpectraClassifier is a user-friendly software designed to fulfil the needs of potential users in the MRS community. The scope of SC is specified; and it is concluded that the results obtained with SC compare well with previous non-automated analysis.

Sandra Ortega Martorell, Ivan Olier, Mª Margarida Julià-Sapé, Carles Arús

Table of Contents

Part I Introduction 4

Part II Main menu 6

1 Main menu options 6
2 Format of the exported files 8

Part III TAB: Classifier design 10

1 Overview 10
2 Importing datasets 10
3 Classes definition 14
4 Feature selection and extraction 14
5 Classifier definition 17
6 Fisher LDA 17

Part IV TAB: Data exploration 20

1 Overview 20
2 Data exploration 20
3 Displaying resulting features 22

Part V TAB: Data visualization 26

1 Overview 26
2 PCA visualization 26
3 Using the visualizer 27

Part VI TAB: Classifier evaluation 32

1 Overview 32
2 Classification results 32
3 Store classifiers info 34
4 Evaluation method 34
5 ROC curve 37

Part VII TAB: Reports 39

1 Overview 39
2 Fisher LDA results 39
3 Fisher LDA probabilities 40
4 Weights matrix 41
5 PCA results 42

Part VIII TAB: Classifier history 45

1 Overview 45

2 Classifier info 45

Part IX References 50

© 2009 GABRMN - Universitat Autònoma de Barcelona

Part I

1 Introduction

Currently available methods for classifying MRS data rely on either commercial (SPSS, SAS), non commercial (R) or home-made programs running over Matlab. Today, multiplatform, easy to use software programs for fast and robust classification of MRS data are scarce.

SpectraClassifier is a java software solution that uses statistical machine learning techniques to design and implement classifiers based on *in-vivo* SV and MV ^{1}H-MRS and high resolution HRMAS.

SpectraClassifier has been developed in java, using well-known multiplatform libraries to carry out specific tasks, such as i) *Weka* [1], used for selecting and extracting features; ii) *JavaStat* [2], used with modifications, to apply Linear Discriminant Analysis (LDA), iii) *StatGraphics* [3], to generate graphs used in classifier results evaluation, iv) *KiNG* (Kinemage, Next Generation) [4] used for three-dimensional visualization.

At the moment, *SpectraClassifier* implements Fisher LDA as the technique of choice to separate two, three or four classes, depending of user needs. *SpectraClassifier* is composed by the following modules: classifier design, data exploration, data visualization, classifier evaluation and reports.

Classifier design tunes the desired inputs for designing the classifier, such as the training datasets, the definition of tumour classes and the selection of relevant features. Three methods, Sequential Forward, Sequential Backward and Principal Components Analysis (PCA) have been implemented for selecting or extracting relevant features. The resulting features or combination of features are used as classifier inputs.

Data exploration allows displaying spectral data, mean and standard deviation for analyzing spectra type population structure and visually comparing spectra.

Data visualization is an up to 3D-latent space visualizer of PCA or LDA results.

Classifier evaluation. An essential part of the life cycle of the classifier development is its validation. *SpectraClassifier* contains several methods for performing this such as: a) Confusion matrix, b) ROC (Receiver Operating Characteristic) curve, c) Graphs showing the statistics of well predicted cases, using test sets, and d) Evaluation methods like cross-validation and bootstrapping with mean and standard deviation results of correctly classified cases, in general and by group.

Reports. The application also allows generating reports with the results obtained.

Main menu

Part II

2 Main menu

2.1 Main menu options

The main menu contains the principal options of the application. These options are divided into the following groups: File, Edit, View, Perform and Help. Following these options are detailed.

File option:

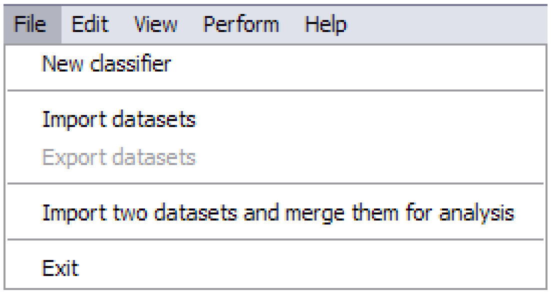

The *File* option allows to create a new classifier, import and export datasets and exit the application. There are two ways to import data, as desired. If you want to work with a spectrum per case you must select the option *Import datasets*, if you want to make a combination of two spectra by case the option *Import two datasets and merge them for analysis* should be used. To export datasets, you will be able to export in a file all cases belonging to those files displayed in the list that you select to export. The application will ask you which list do you want to export (*Imported datasets*, *Training datasets*, *Testing datasets*).

Edit option:

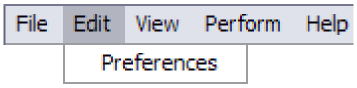

The *Preferences* option from Edit allows to configure certain aspects of the interface, like the appearance (also called *look and feel*) and the position of the tabs. When the Preferences option is clicked, the application displays a window to modify the already mentioned configuration. See the following figure.

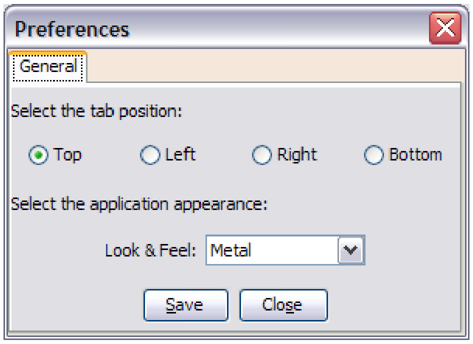

The Save button is used to accept changes made and the Close button to leave this window.

© 2009 GABRMN - Universitat Autònoma de Barcelona

View option:

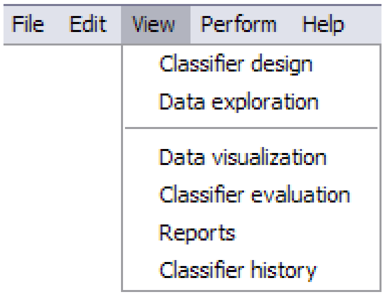

The *View* option allows the user to switch tabs.

Perform option:

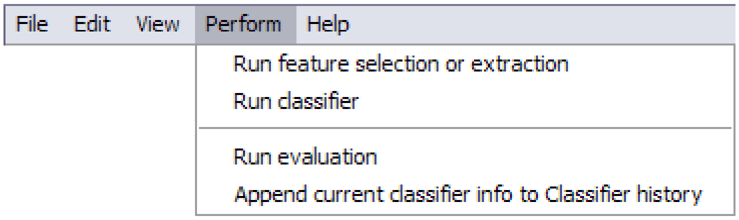

The *Perform* option groups the principal operations of the application, like *Run feature selection or extraction*, *Run classifier*, *Run evaluation*, and *Append current classifier info to Classifier history*

Help option:

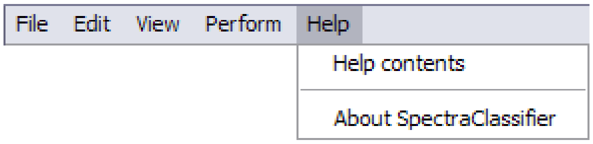

The *Help* option allows the user to open the *Help contents*, and the *About SpectraClassifier* to see info related to the version and release date of the application, etcetera. See the following image.

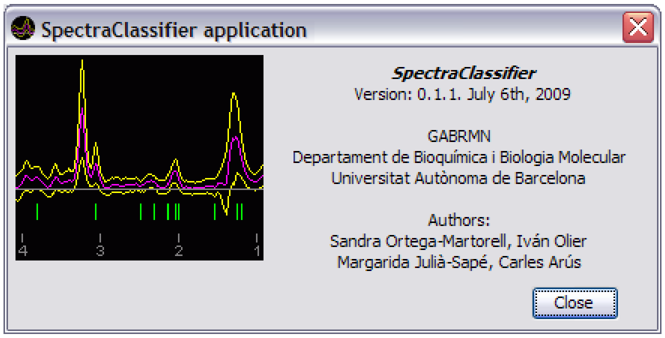

© 2009 GABRMN - Universitat Autònoma de Barcelona

2.2 Format of the exported files

The exported file follows the XML schema developed for storing all the information needed for a dataset. See the following figure.

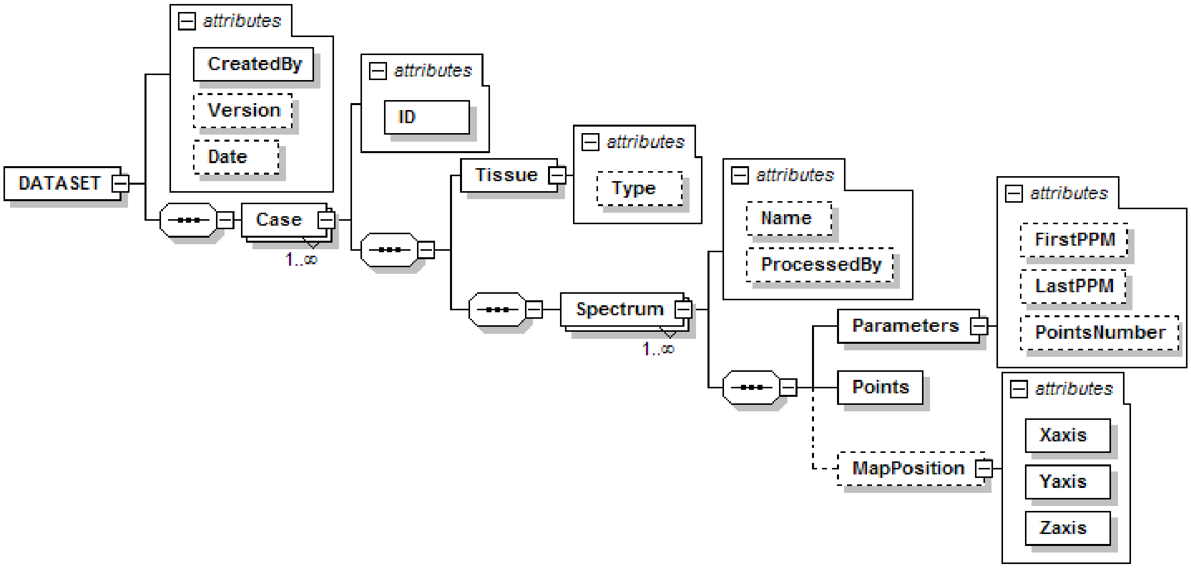

XML Schema developed to describe a dataset.

As shown in the figure, the global node is DATASET, with attributes *CreatedBy*, *Version* and *Date.* The *CreatedBy* and *Version* attributes express which application built this file and with what version, in this case the value for created by is always *SpectraClassifier.* The *Date* is just the date the file was exported. *Version* and *Date* are non-mandatory.

Every data set node will have one or more *Case* nodes. A *Case* node has an *ID* attribute for the identification of the case, and a sequence of nodes like *Tissue* and *Spectrum*. A case has only one *Tissue* node and one or more *Spectrum* nodes. The *Tissue* node has a *Type* attribute (non-mandatory). Every *Spectrum* node has two mandatory child nodes: *Parameters* and *Points*; and one non-mandatory node: *MapPosition.* The *Parameters* node has three non-mandatory attributes like *PointsNumber* for the number of points of the spectrum, *LastPPM* for the last PPM and *FirstPPM* for the first PPM. The *Points* node is used to store the spectrum quantitative data, i.e. the intensity value of each point in the frequency domain, and the *MapPosition* node is used to store the x-y position of each spectrum in each MV grid. Dashed lines are used to indicate non-mandatory elements.

Part III

3 TAB: Classifier design

3.1 Overview

When the ***SpectraClassifier*** is launched the first thing you see is the **Classifier design tab** that allows you to introduce the needed information to make a classifier.

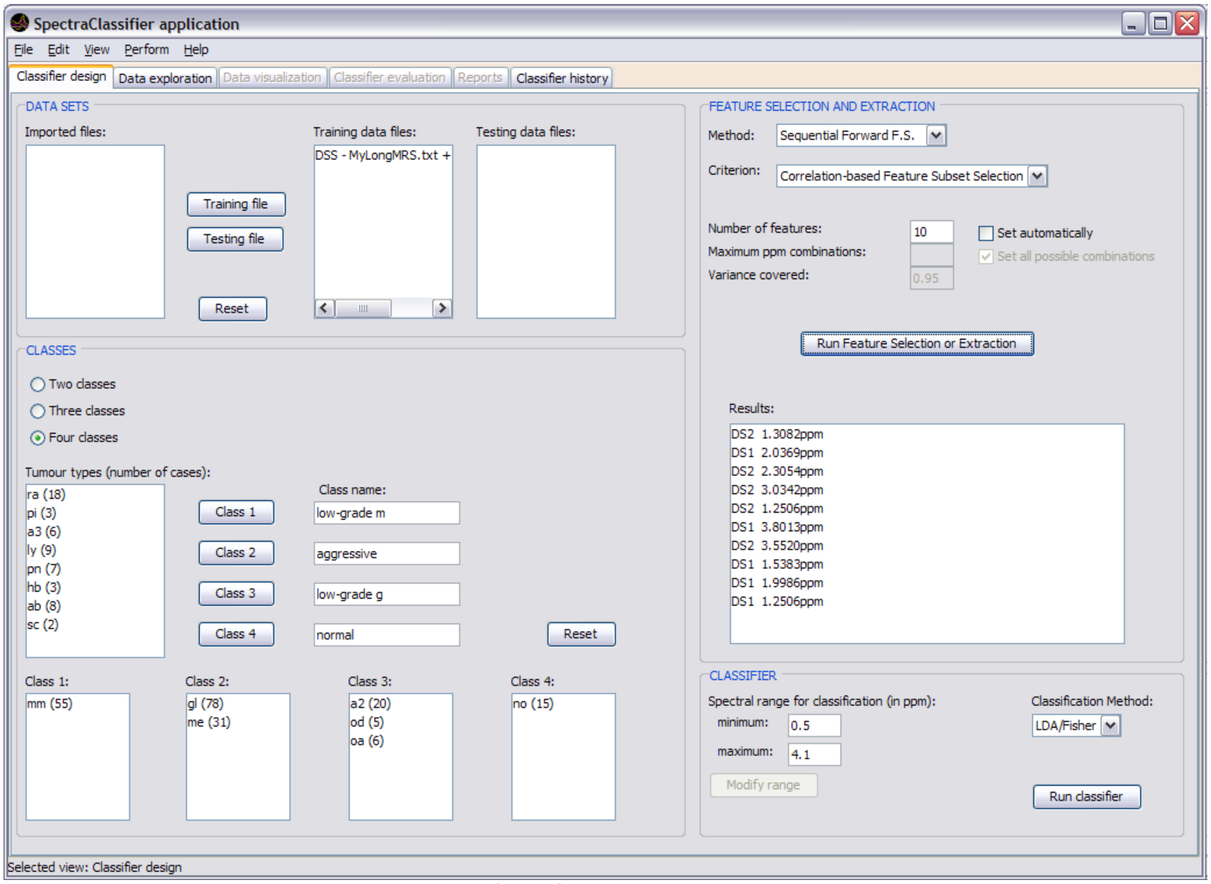

Classifier design tab

3.2 Importing datasets

The first thing to do is to import datasets. There are two ways to import data, as desired. If you want to work with a spectrum per case you must select the option *Import datasets* (File/Import datasets), if you want to make a combination of two spectra by case the option *Import two datasets and merge them for analysis* should be used (File/Import two datasets and merge them for analysis).

Importing a spectrum per case

There are some validations for importing datasets:

1) If you import a file that does not contain the tumour type information, the application is going to ask for it, (see the following figure).

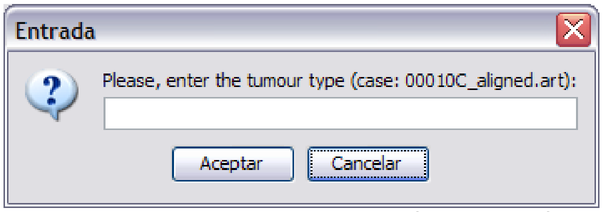

Importing a case without the information of the tumour type (from Classifier design tab)

Note: all cases having the tumour type information (either because was read in the file, or because the user entered it) may be used for training or testing, those that are left empty because the type of tumor is unknown, may only be used as test cases.

2) If you import a file that does not contain the information of the spectral range in ppm, the application is going to ask for it, (see the following figure):

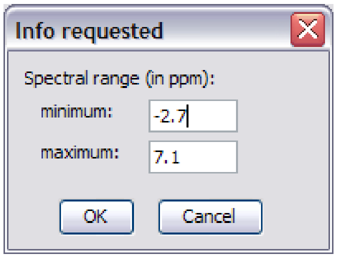

Importing a case without the information of the spectral range (from Classifier design tab)

3) Once you imported a case, the application is going to use the ppm range entered as the reference for the following files to import. So, if you enter a new case with a range of ppm that does not correspond with the previously entered, the application will inform you that, and will not allow you to enter this case. If the new case does not contain the range information, the application is going to ask you if you want to used the range entered previously (the one of the reference). If you say yes, the case will be entered with this range, if you say no, you will not be allowed of enter this case in the group of cases being created.

4) Another validation is related with the number of points of the spectra. All cases should have the same number of points, and the number of points of the first case entered is going to be used as the reference for the following files to import.

Importing two spectra per case

If you select to *Import two datasets and merge them for analysis,* the application is going to show a window to allow you to enter the two datasets (see the following image). *Dataset 1* and *Dataset 2* buttons can be used to select the files you want to import. When you press the *OK* button, the application will merge every spectrum of the first dataset with the corresponding spectrum of the second one. The allowed file extensions of both datasets do not need to be the same, but the number of cases in both has to be the same.

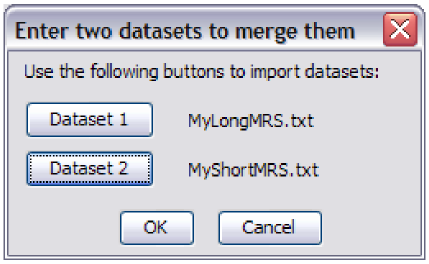

Enter two datasets to merge them (from Classifier design)

To import two sets, some validations must be met in addition to the validations for each dataset separately (mentioned below in Importing a spectrum per case):

1) The number of cases has to be the same in both datasets.

2) The number of points of every spectrum has to be the same in both datasets.

3) The name of the case, the tumour type, and the spectral range in ppm, are always taken from the Dataset 1 (even if dataset 1 does not have this information and Dataset 2 does). So, if you enter in Dataset 1 a file that does not contain this information, the application is going to ask for it (as in Importing a spectra per case).

If you enter a dataset that contains two spectra per case as Dataset 1, the application is going to ask you if you want to use both spectra merged. If you say *Yes*, then the application is going to ask you if you want to use them in the same order that they appear (it means that the first spectra is for Dataset 1 and the second for Dataset 2). If you say *No* to the option of using both spectra merged, then the application understands that you want to select only one spectrum per case, and then it is going to ask you which of those spectrum do you want to use (if the first or the second one).

Allowed extensions:

For importing dataset files, the preferable format is the XML with the structure described before. It can be used for the three types of MRS data allowed by *SpectraClassifier*. Other formats can also be used to import dataset files, according to the type of MRS data:

1. In-vivo SV data, usually with a low number of points per spectrum (512-2048):
   1.1. File with extension .txt or .art in the INTERPRET [5] canonical format, with 512 points in the [7.2; -2.8] ppm range, which only contains the information of one spectrum in one row. Similarly, files with extension .dat, exported with SPSS or similar, and composed by rows of 514 tokens, where the first row is columns labels (not used in *SpectraClassifier*), and the rest of rows correspond to cases (similar to the INTERPRET canonical format), having the following information each: identifier of the class, identifier of the case, and 512 points of the spectrum.
   1.2. File with extension .txt, processed and exported using the Magnetic Resonance User Interface package (jMRUI) [8]. It is composed by a header and a four-column matrix of data. The header is partially used by *SpectraClassifier*, because it contains the number of points of the spectrum *(PointsInDataset)*, and the information that allows inferring the spectral range (*SamplingInterval* and *TransmitterFrequency*). From the data matrix, only the third column (fft(real)) is read by *SpectraClassifier*.

2. In-vivo MV data, also with a low number of points per spectrum (512-2048), but with a large number of spectra per acquisition (n x n). *SpectraClassifier* treats each acquisition as one dataset:
   2.1. File with extension .bsp, that corresponds to data pre-processed with 3D Interactive Chemical

Shift Imaging v1.9.10 (3DiCSI) [9], and exporting the data in ASCII format [10]. It has the following structure: first row for the name of the set (not used in *SpectraClassifier*), line-break, Number of voxels: (a number), line-break, Number of points per voxel: (a number), line-break, Voxel Index: (with the information of the location of each voxel in a map, it is not used by *SpectraClassifier*), line-break, and then two columns with Real and Imaginary data.

3. High resolution data, usually with a large number of points per spectrum (16-32 K points).
   3.1. File with extension .txt, for HRMAS. The original file having been processed with TopSpin [11] or similar and exported as text file. The number of points accepted is variable; the most commonly used are from 1600 to 3200, with a [4.5; 0.5] ppm range. Each file only contains the information of one spectrum in one column.

The pre-processing tasks needed for imported files are out of the scope of *SpectraClassifier*, so they have to be carried out before using this software, including adjustments of sweep width and number of points if spectra from different manufacturers are to be used. On the other hand, all imported datasets, training and testing sets, regardless of its original format, can be exported in the XML file format described before.

Setting datasets as training or testing:

Imported datasets could be used as training or testing sets. After an imported file is chosen (see figure below) you can pick between **Training file** button and **Testing file** button to indicate that all cases in this file are going to be for training or testing, respectively. The **Reset** button is to set all inputs as default and restart the design of the classifier.

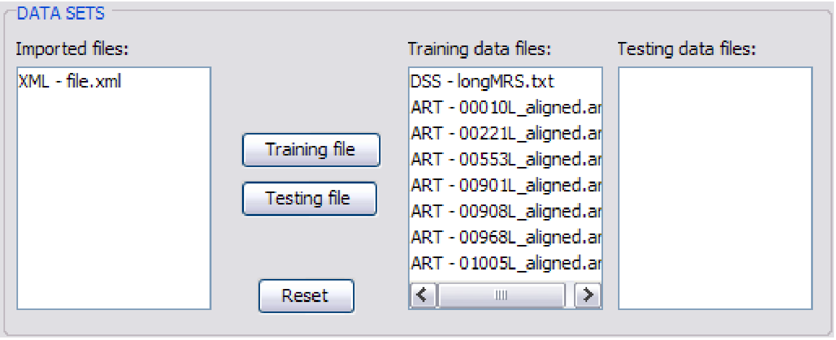

Creating data sets (from Classifier design tab)

Note that the names of the imported data sets are formed by the type of the data set followed by the name of the file. The following list shows all types of the data set used.

Type	Description
DSS	Text file from the INTERPRET decision support system [5]
jMRUI	Text file from jMRUI
HRMAS	HRMAS text file
BSP	BSP file from Biospec spectrometers (Bruker)
ART	ART file from the INTERPRET data manipulation software [5]
DAT	DAT file from SPSS-DAT
XML	XML file with GABRMN format

© 2009 GABRMN - Universitat Autònoma de Barcelona

3.3 Classes definition

The figure below shows three radio buttons to select the number of classes, you can select among two, three or four classes. After setting imported files as training dataset, the **Tumour types** list box is filled in with all the types read from training cases, so you can select them to create classes or groups. To do that you should mark the tumour types and press the button of the class you need, the tumour type is going to be removed from the Tumour types list box and is going to be added in the list box of the selected class. The **Class name** text is optional and the **Reset** button is to set classes as default.

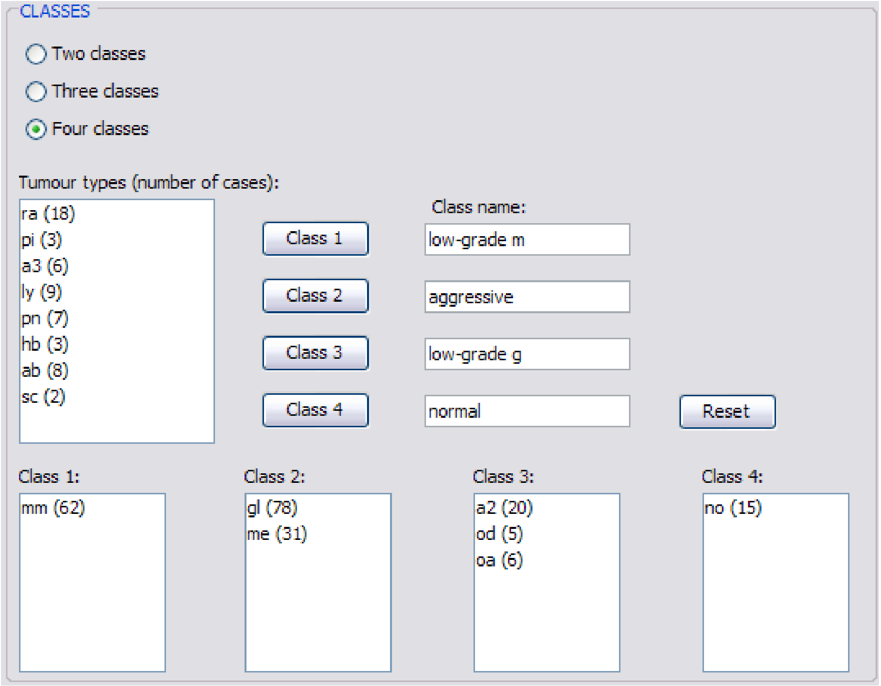

Selecting classes (from Classifier design tab)

Note that the tumour type preselected abbreviature is followed by a number in parenthesis. This is the number of cases in the training dataset that are labelled with this tumour type.

3.4 Feature selection and extraction

Once the training data set is imported, and the classes of tumour types are indicated, you can do the feature selection or the feature extraction, according to the selected method. In general, the following figure shows the selectable fields. Note that in the Results box is indicated the dataset where each relevant feature belongs.

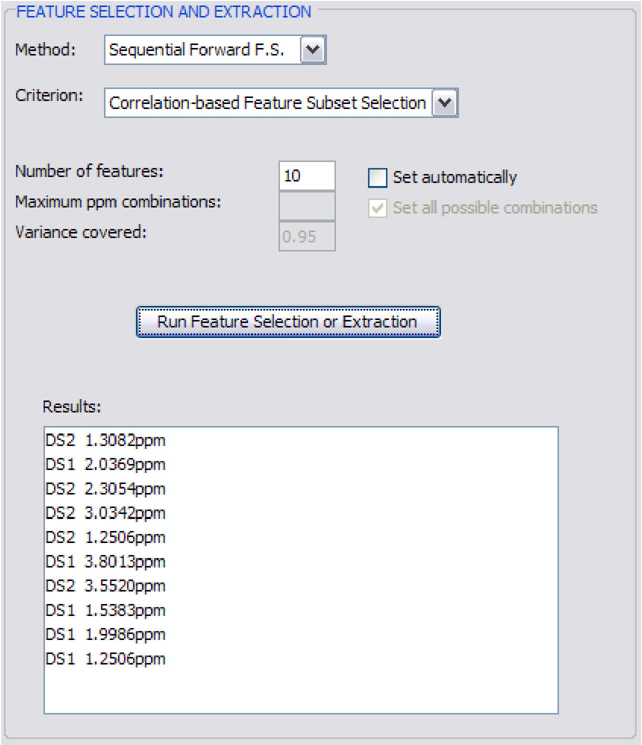

Feature selection and extraction (from Classifier design tab)

If the **Method** is "Sequential Forward F.S." or "Sequential Backward F.S.", you will be able to set the **Criterion** and the **Number of features**. Both methods are for feature selection. If the **Method** is PCA (see the following figure), you will be able to set the **Number of features**, the **Maximum ppm combination** and the **Variance covered**.

The **Number of features** represents the desirable number of resulting features after running the **Run Feature Selection or Extraction** button. If this number of features is set automatically, then the number of resulting features will be the maximum selected by the system to optimize its output.

The **Maximum ppm combination** is used in Principal Components to specify the maximum number of attributes to include in transformed attribute names. You can check the **Set all possible combination** radio button to include all.

The **Variance covered** box tells the system to retain enough principal components to account for this proportion of variance in the original data. The default value has been set to 0.95.

The following figure shows the results of applying a PCA that retained 5 principal components, with 2 combinations of ppm, and retained 95% of variance.

© 2009 GABRMN - Universitat Autònoma de Barcelona

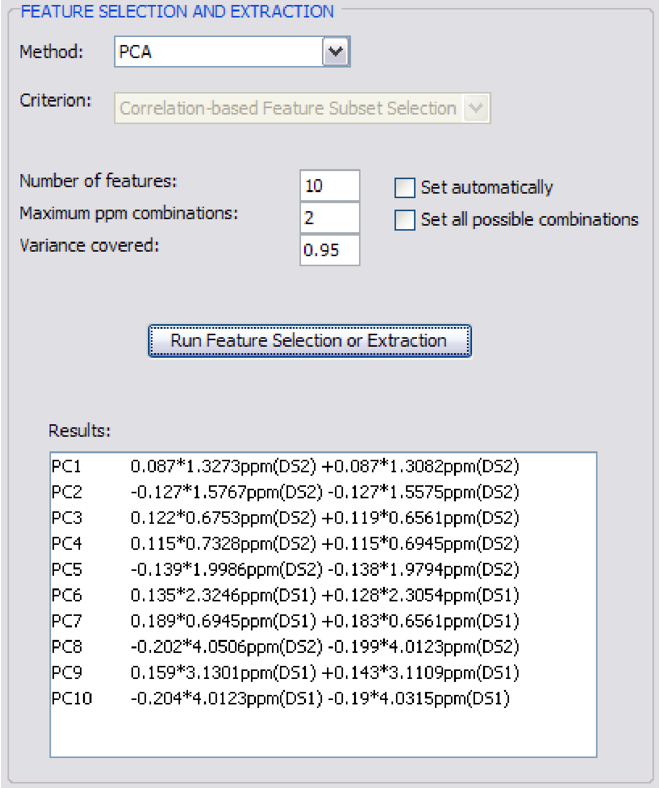

PCA (feature extraction) (from Classifier design tab)

Inside the process of feature selection of "Sequential Forward F.S." or "Sequential Backward F.S." methods, there is an evaluation of these features by means of the *Correlation-based Feature Subset Selection for Machine Learning* technique implemented in the class *CfsSubsetEval* of Weka [1]. This class evaluates the worth of a subset of attributes by considering the individual predictive ability of each feature along with the degree of redundancy between them. Subsets of features that are highly correlated with the class while having low intercorrelation are preferred.

The PCA method performs a principal components analysis and transformation of the data, used in conjunction with a *Ranker* search. Dimensionality reduction is accomplished by choosing enough eigenvectors to account for some percentage of the variance in the original data [default 0.95 (95%)]. Attribute noise can be filtered by transforming to the PC space, eliminating some of the worst eigenvectors, and then transforming back to the original space. The *Ranker* ranks attributes by their individual evaluations.

Some validations for obtaining relevant features

1) As in Tate et al. [5], if the user select a number of features greater than n/3, where n is the number of cases in the smallest group of the training set, the application is going to show a warning for possible data overfitting (see figure below). If the n/3 value is less than the number of classes-1, the suggested value will be the number of classes - 1.

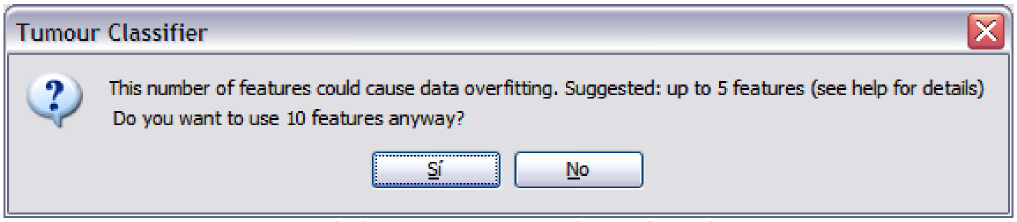

Number of features warning (from Classifier design)

2) Fields like *Number of features*, *Maximum ppm combinations* and *Variance covered* only accept the input of numeric values.

3) The *Number of features* should be less than the number of points of each spectrum in the dataset.

3.5 Classifier definition

The **Classifier** panel (see figure) of the **Classifier design** tab allows to change the **Spectral range for classification (in ppm)**, setting the minimum and maximum value. You can also select the **Classification Method**.

The **minimum** and **maximum** values of the spectral range for classification are 0.5 and 4.1 by default because this is the region of interest where the resonances of the main metabolites arise and where the contribution of the residual water is expected to be minimal [6]. But you can change these values by those you consider most appropriate to build the classifier.

The available classification method at the moment is Fisher LDA, which calculates a set of linear discriminants and obtains the predictions for the data.

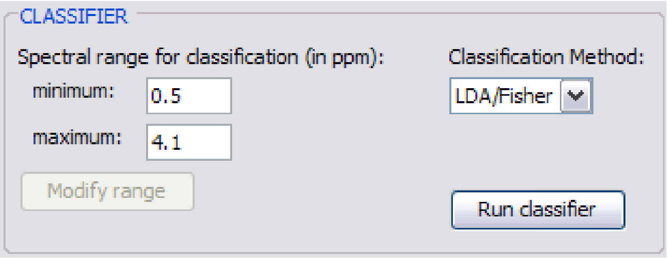

Classifier (from Classifier design tab)

After setting all information (data sets, classes and features) you can press the **Run classifier** button to calculate the classifier.

3.6 Fisher LDA

At the moment, SC uses Fisher LDA as the technique of choice for distinguishing cases between two, three or four classes. Each class could be a tumour type, various tumour types forming a super-class, normal tissue, etc. Fisher LDA is a fundamental and widely used technique, that provides a reasonably way of reducing the dimensionality of the problem.

For the implementation of the Fisher LDA classification method, the *JavaStat* library was used. JavaStat, implemented using Java, is an open-source, platform-neutral library for performing basic statistics. The Discriminant Analysis class implemented in JavaStat mainly follows the formulae in [7] (see Chapter 11.3 and 11.6). For its used inside *SpectraClassifier*, a few modifications have been made in order to allow the use of more than 4 features. Below you can see this modification:

© 2009 GABRMN - Universitat Autònoma de Barcelona

Line 586 of "multivariate/DiscriminantAnalysis.java", rewrite the for sentence to:

```
for (int m=1; m < groupIndex.length; m++ )
```

instead of:

```
for (int m=1; m < (eigenValues.length-1); m++ )
```

TAB: Data exploration

Part IV

4 TAB: Data exploration

4.1 Overview

Data exploration is the second tab of the application. You can use it to plot cases, to plot the mean and the standard deviation of data sets, and to display the selected features on **Classifier design** tab (if the used method is "Sequential Forward F.S." or "Sequential Backward F.S.").

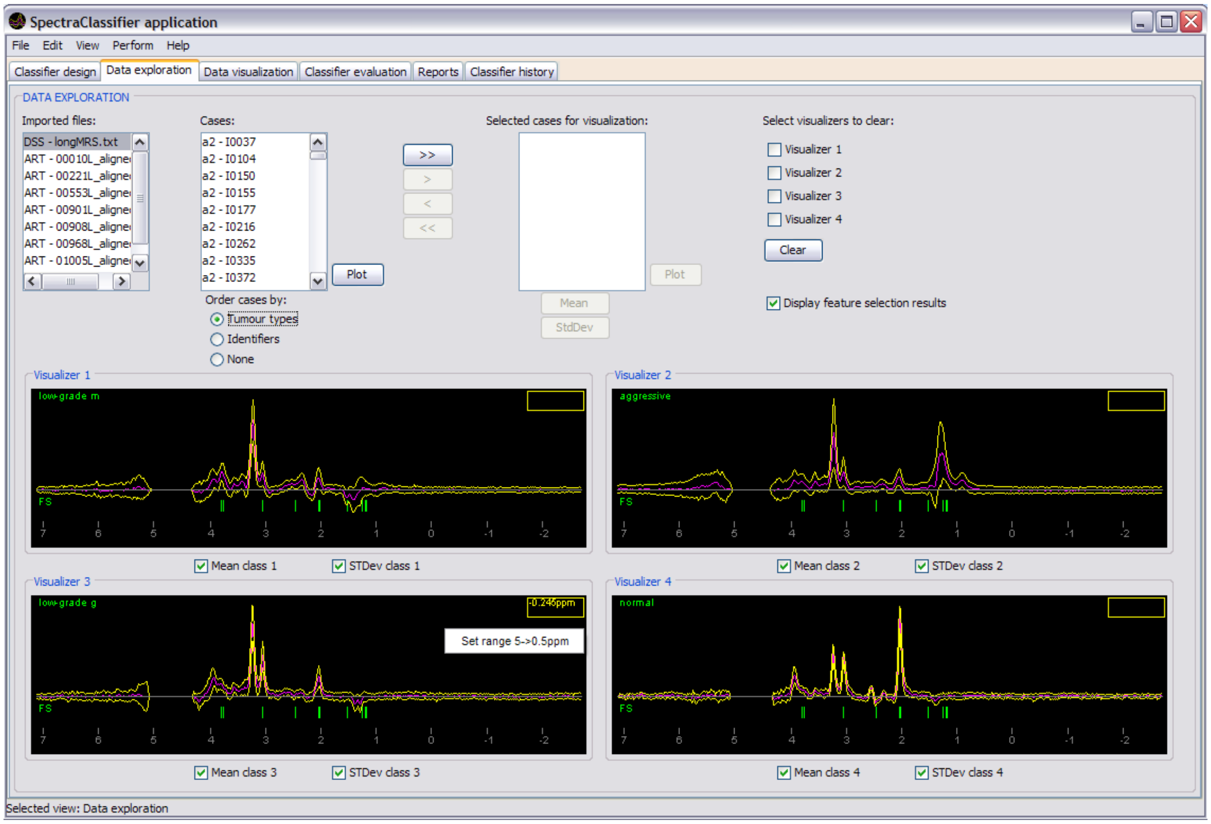

Data exploration tab

4.2 Data exploration

Plot cases:

To plot cases, mean and standard deviation of data sets, first you have to import a data set. All imported data sets files will be listed in the **Imported files** list box (see Imported files and Cases figure). If you select one of the listed files, all the cases (tumour type and identifier) will be listed in the **Cases** list box.

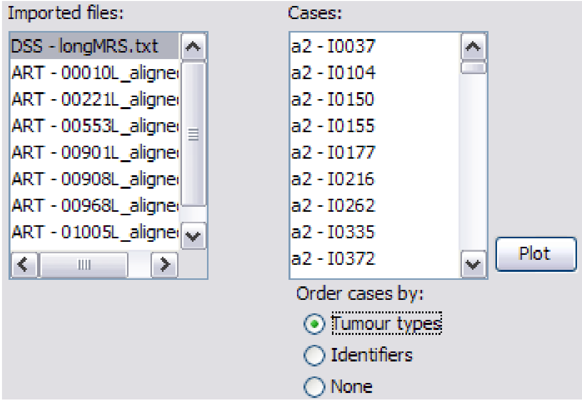

Imported files and Cases (from Data exploration t

You can sort the cases of the Cases list box by tumour types or identifiers. The **None** option leaves data unsorted, just in the order of appearance in the imported file. For example, the figure above shows the cases of the dataset *DSS-longMRS.txt* ordered by tumour types.

When you press the **Plot** button, you will have to indicate in which visualizers you want to plot the selected cases. You can select one or more visualizers at the same time. See the following figure.

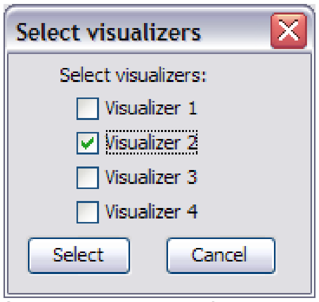

Select visualizers (from Data exploration tab)

Plot mean and standard deviation:

You can select specific cases from the **Cases** list box and add them to the **Selected cases for visualization** list box (see the following figure).

© 2009 GABRMN - Universitat Autònoma de Barcelona

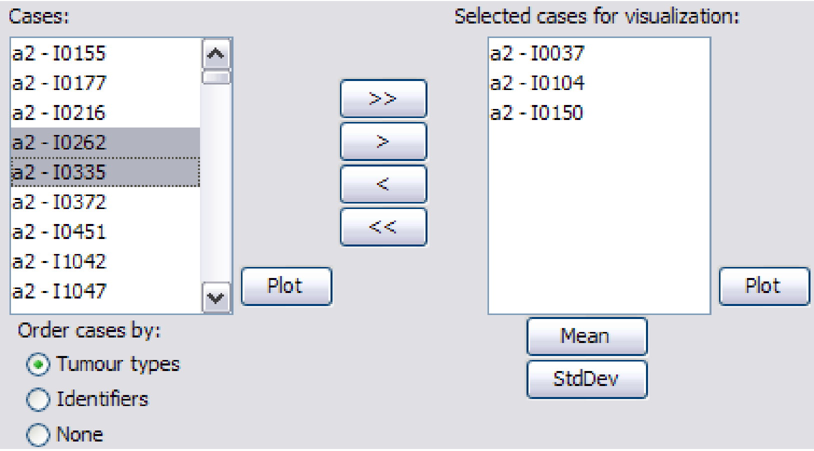

Cases and Selected cases for visualization list boxes.

For plotting mean and standard deviation you should add the cases to the **Selected cases for visualization** list box. Then press the **Mean** or **StdDev** button, respectively. You can even plot individual cases from the Selected cases for visualization list box, using the right bottom option **Plot**.

Use buttons in the middle as follows:

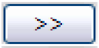

Add all from **Cases** list box to **Selected cases** list box.

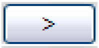

Add marked cases in **Cases** list box to **Selected cases** list box.

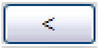

Remove marked cases from **Selected cases** list box and add them to **Cases** list box.

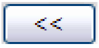

Remove all cases from **Selected cases** list box and add them to **Cases** list box.

4.3 Displaying resulting features

Display feature selection results

If the used methods for feature selection are "Sequential Forward F.S." or "Sequential Backward F.S.", you can display the selected features on **Classifier design** tab in the Data exploration tab's visualizers. To do that, you should check the **Display feature selection results** box (see next figure). If you choose the PCA method, then the **Display feature selection results** box will be disabled.

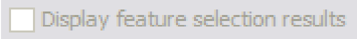

Display features disabled (from Data exploration tab)

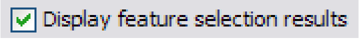

Display features enabled (from Data exploration tab)

After checking **Display feature selection results** box, accordingly to the number of classes, a number of visualizers will show the selected features in each class. (See the following images). Note that you can see the mean (the pink plot), the standard deviation range (the yellow plots) and the selected features (green lines). The numbers in grey are in ppm and the yellow box to the right indicates the exact ppm number where the mouse is located in the visualizer. You can uncheck the **Mean** or **STDev** boxes to hide those plots.

© 2009 GABRMN - Universitat Autònoma de Barcelona

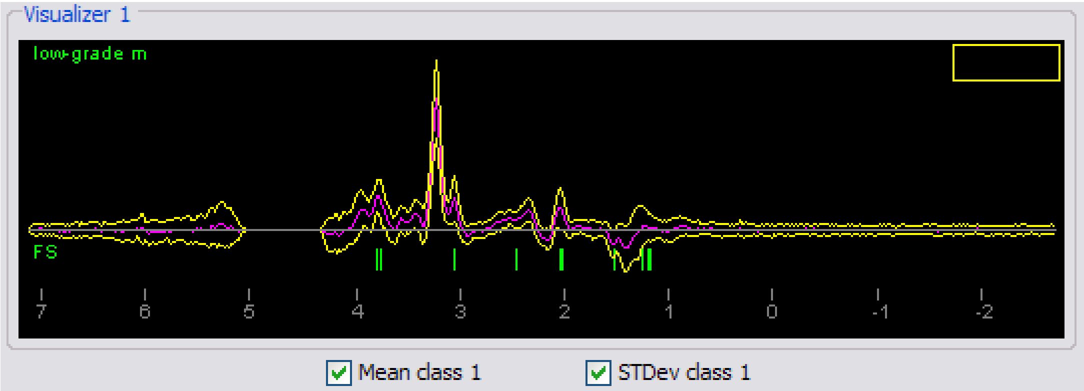

Details of one of the visualizers (from Data exploration tab)

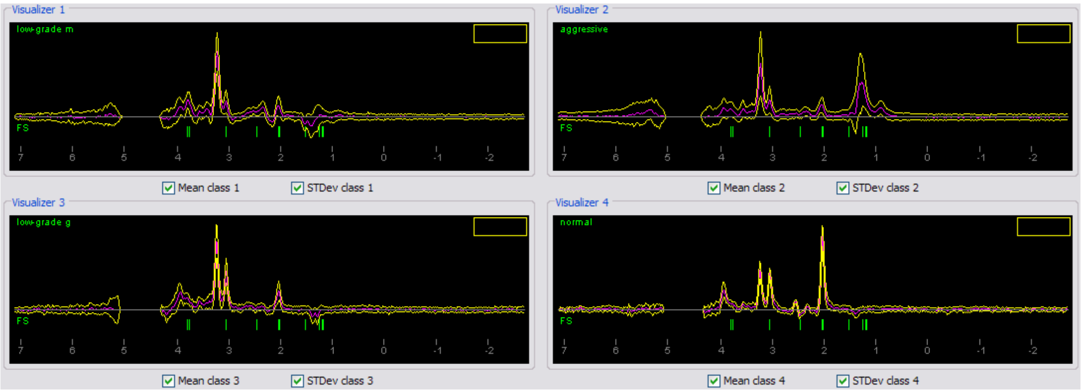

All the visualizers (from Data exploration tab)

Change visualization range

You can use the right button of the mouse to change the visualization range. There are two options implemented: *Set full range* and *Set range 5 -> 0.5 ppm.*

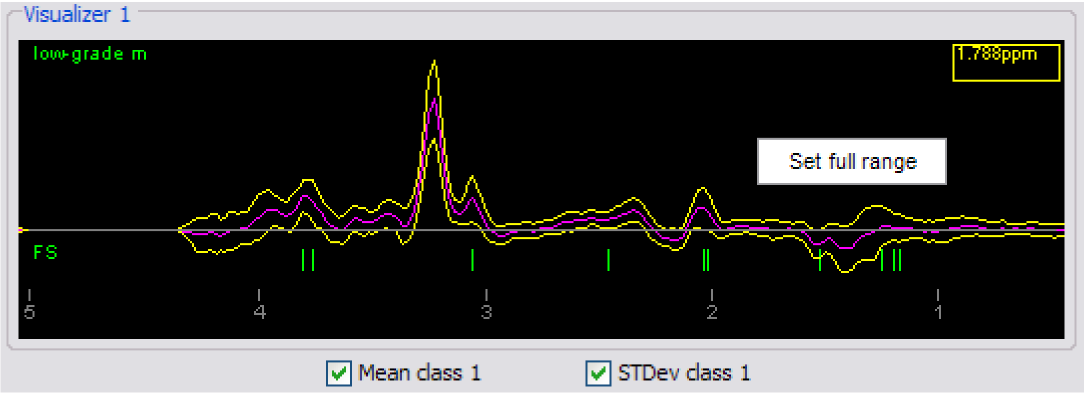

Set full range option (from Data exploration tab)

Clear

You can clean the visualizers you need using **Select visualizers to clear** as shown below:

© 2009 GABRMN - Universitat Autònoma de Barcelona

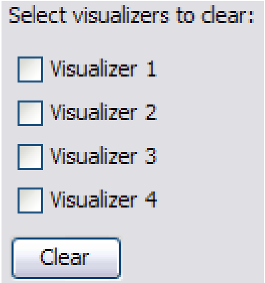

To clear visualizers (from Data exploration tab)

Part V

5 TAB: Data visualization

5.1 Overview

Data visualization is the third tab of the application. You can use this tab to visualize resulting data from PCA or Fisher LDA. The following figure shows a Fisher LDA classifier visualization for the following brain tumor classes: low-grade meningioma, aggressive, low-grade glial and normal. The *KiNG* (Kinemage, Next Generation) library was used for three-dimensional visualization.

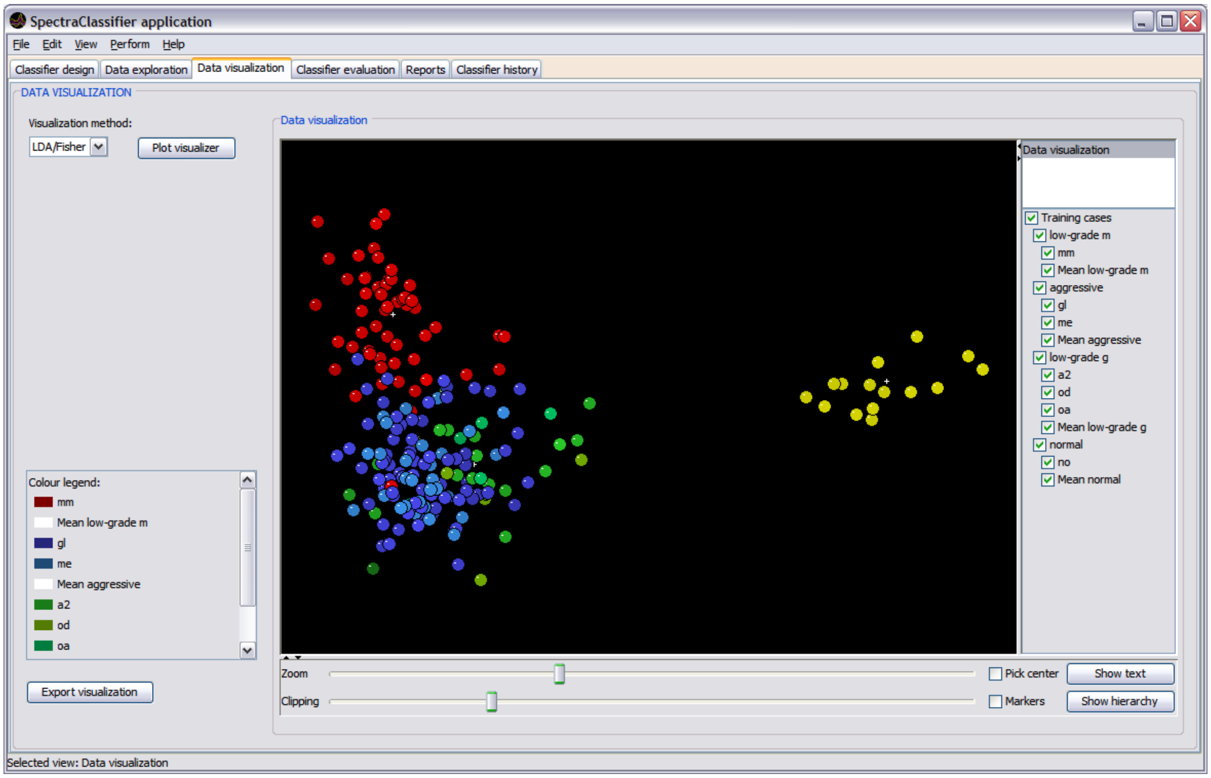

Data visualization tab

5.2 PCA visualization

To visualize resulting data from PCA, first you have to select PCA in the **Visualization method** combo box (PCA is in this combo box only if you make PCA before in **Classifier design** tab). Then you should indicate the pair of components to be set as horizontal and vertical axes. To set PC as axis, you should select one of the listed in the **PCA** list box and press **Set as horizontal axis** or **Set as vertical axis** button. You can not select the same component to be both the horizontal and vertical axis. As you can see in the following image, the selected PCs will be shown on the bottom right of the box.

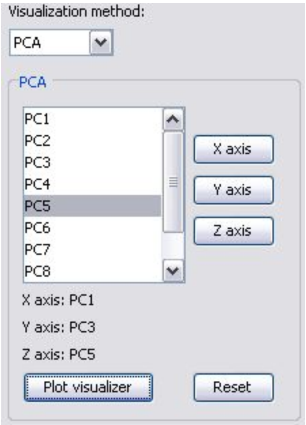

Selecting the component to visualize (from Data visualization tab)

The visualization will be as follows:

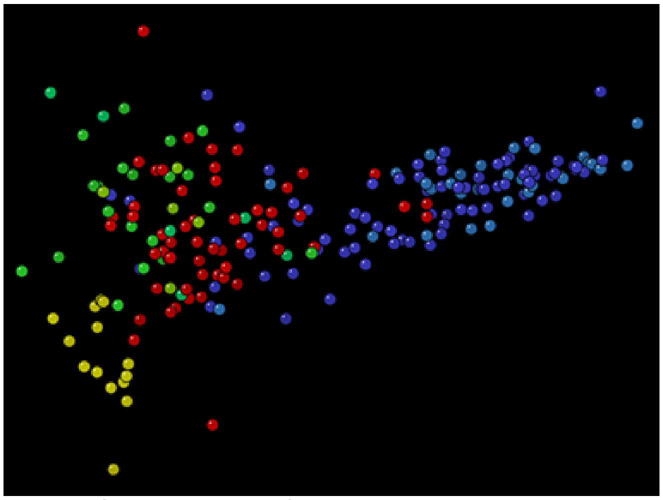

PCA visualization (from Data visualization tab)

5.3 Using the visualizer

The *Data visualization panel* is a 3D illustration of the corresponding point in the space of each case. In a 3D representation, the best way to take advantage of this visualization is by rotating it and twisting it around. Just click the mouse near the center of the graphic panel and slowly drag right or left, up or down. If you get lost or disoriented, you can re-plot the visualization by means of the *Plot visualizer* button to start all over again.

All tumour types involved in the classifier are grouped into two main groups: *Training cases* and *Testing cases*. Testing cases group appears only if you enter a test set to the application. Both groups are divided into subgroups, each of them for a class. The groups and subgroups appear in the button panel, to the right of the graphic area. Each one has a check-button to turn it on or off. Groups that are on (visible), usually have a check mark or an X in their box; a blank button means that the group is hidden.

By clicking on cases with the left button of the mouse, you will be able to identify cases. The identifier of the case will appear in the bottom left of the graphics area. Furthermore, the distance from this point to

© 2009 GABRMN - Universitat Autònoma de Barcelona

the last one you clicked will also be displayed. For keeping track of which case is selected, markers can be displayed. Two markers are displayed normally. The checkbox *Markers* is just below the graphic area, with the *Pick center* checkbox.

You can make any case in the visualization to be the center. The center case will be in the middle of the graphic area, and the visualization will rotate about that case. There are several ways to set the center: you can hold the Shift key while you click the case, or use the right button of the mouse if you have one. You can also press the *Pick center* button below the graphics area and then click on the desired case.

The Data visualization panel allows you to zoom in to see small details in the visualization. Use the Zoom slider, below the graphic area, to control how far you zoom in. You can click the mouse right or left of the knob for small movements or click the knob and drag it for larger ones. A small motion is usually all that is needed. For easier access, one can hold Shift and drag (or drag using the right mouse button) in the graphic windows. Dragging down zooms in; up zooms out. The up/down arrow keys and mouse wheel (Java 1.4 and later only) also control zooming.

In a 3D representation, objects that are too near the viewer, or too far away, are not displayed; otherwise, zooming in would result in a useless superposition. The depth of the clipping slab can be adjusted using the slider along the bottom, or by dragging side-to-side with the right mouse button.

The following figure shows some of the basic tools to interact with the visualization, explained above.

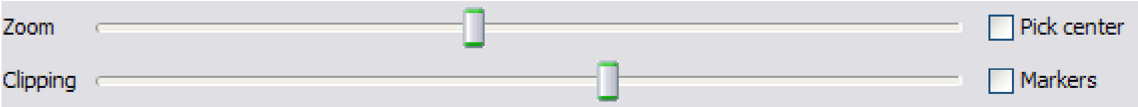

Some basic tools to interact with the visualization (from Data visualization tab)

Show hierarchy button can help to modify the current visualization. The structure can be rearranged by cutting, copying and pasting elements; creating new elements and deleting unneeded ones; and reordering elements (using the Up and Down commands). Play with these commands, and their operations will soon become obvious. Also, the properties of individual elements can be adjusted, which allows them to be renamed. Each element can also be toggled on and off. You can change the colors and the size of elements (see next figure).

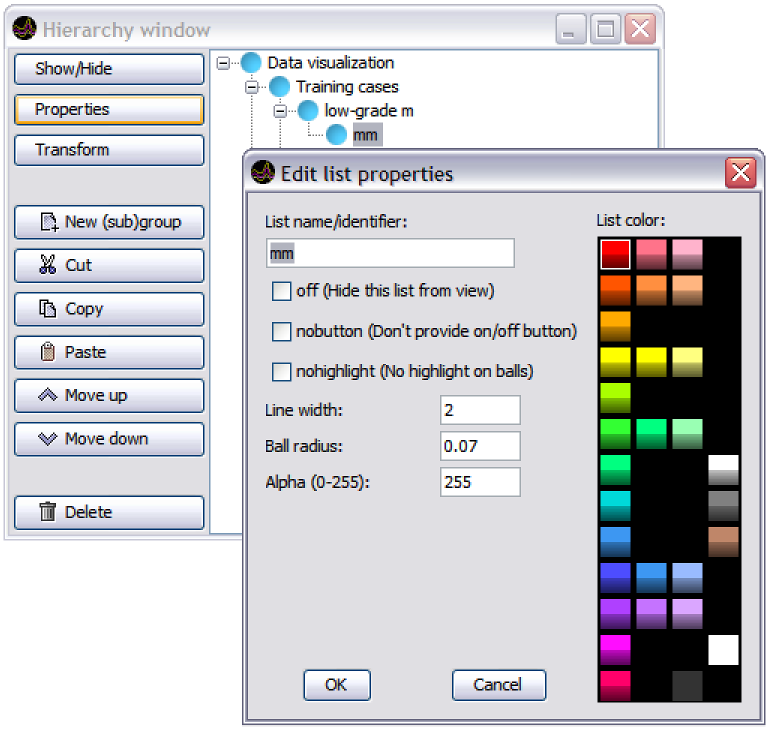

Hierarchy window (from Data visualization tab)

To set white the background of the visualizer, just select the *Data visualization* element in the *Hierarchy window* (see previous figure) and click *Properties*. Then, check the option *whiteback* and press OK. To return to a black background, then uncheck the *whiteback* option.

On the left of the *Data visualization panel*, there is a *Colour legend* of the tumour types already plotted.

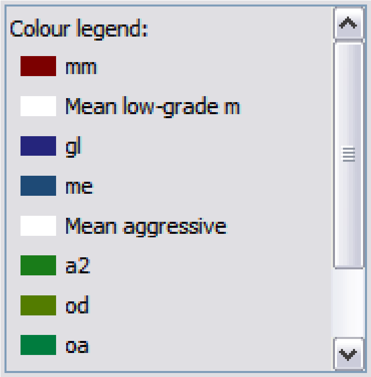

Colour legend (from Data visualization tab)

The *Export visualization* button can be used to export the current plot view of the *Data visualization panel* as a standard image file. This button is located below the *Colour legend*, and on the left of the *Data visualization panel*.

As the original version of Fisher LDA does not assume any probability distribution to define the model, the limitation of Fisher LDA for estimating the probability of a case of belonging to a class, has been overcome by approximating the resulting projections through spherical Gaussian distributions, one for each class. The centre of each distribution has been assumed as the class mean estimated from data

© 2009 GABRMN - Universitat Autònoma de Barcelona

and the standard deviation common to all. Therefore, the probability of membership of every case to each class is estimated applying the Bayes' theorem over these distributions:

$$P(\omega_i \mid X) = \frac{p(X \mid \omega_i)}{\sum_{j=1}^{c} p(X \mid \omega_j)}$$

where ω_i are each of the classes; X is the projected case after applying LDA; $p(\omega_i|X)$ is the posterior probability; $p(X|\omega_i)$ is the normal distribution in the class ω_i, with the mean of the projected data belonging to that class, and the common variance estimated for the entire set of projected data. The method assumes equal prior probability for each class, as LDA.

Classes boundaries are going to be displayed in case of a 2 or 3 -classes classifier visualization with Fisher LDA. The mean of the classes are also going to be displayed as crosses.

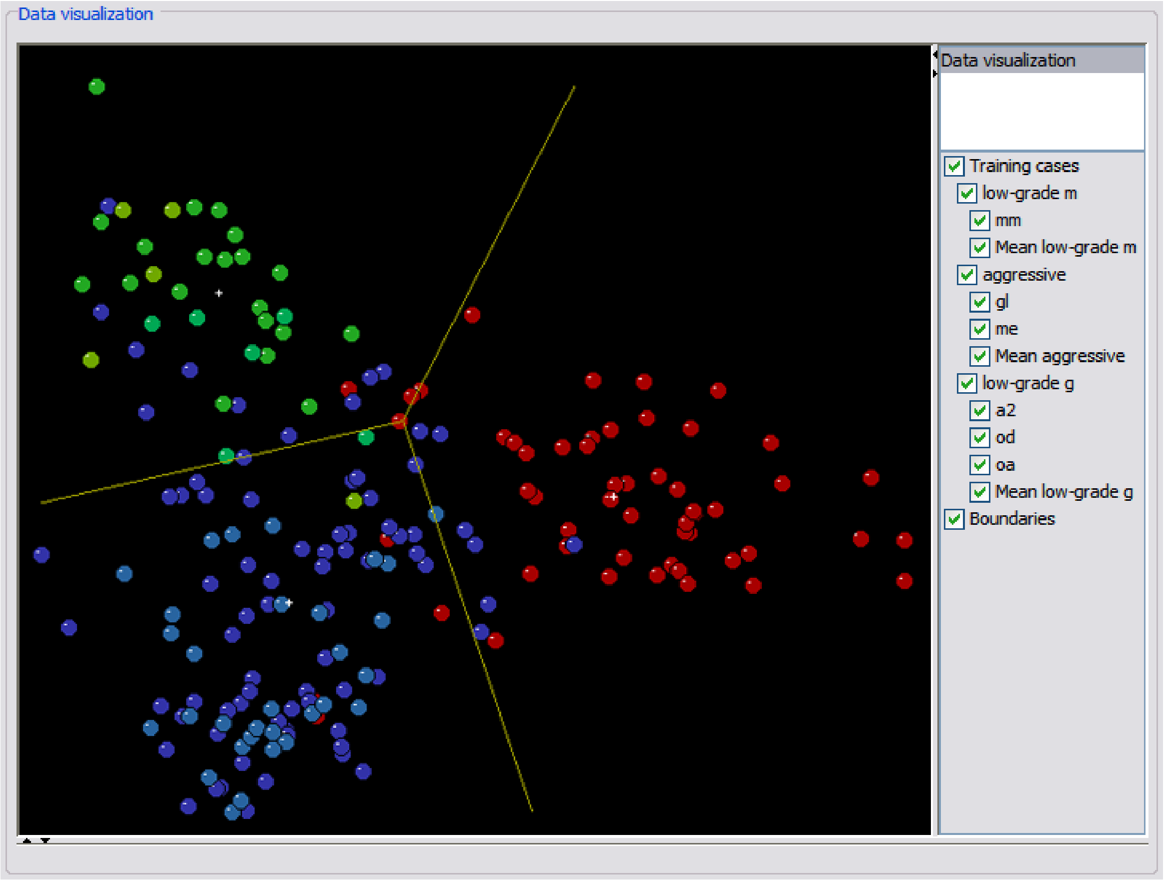

Visualization with boundaries (from Data visualization tab)

Part VI

6 TAB: Classifier evaluation

6.1 Overview

Classifier evaluation tab is the fourth tab of the application.

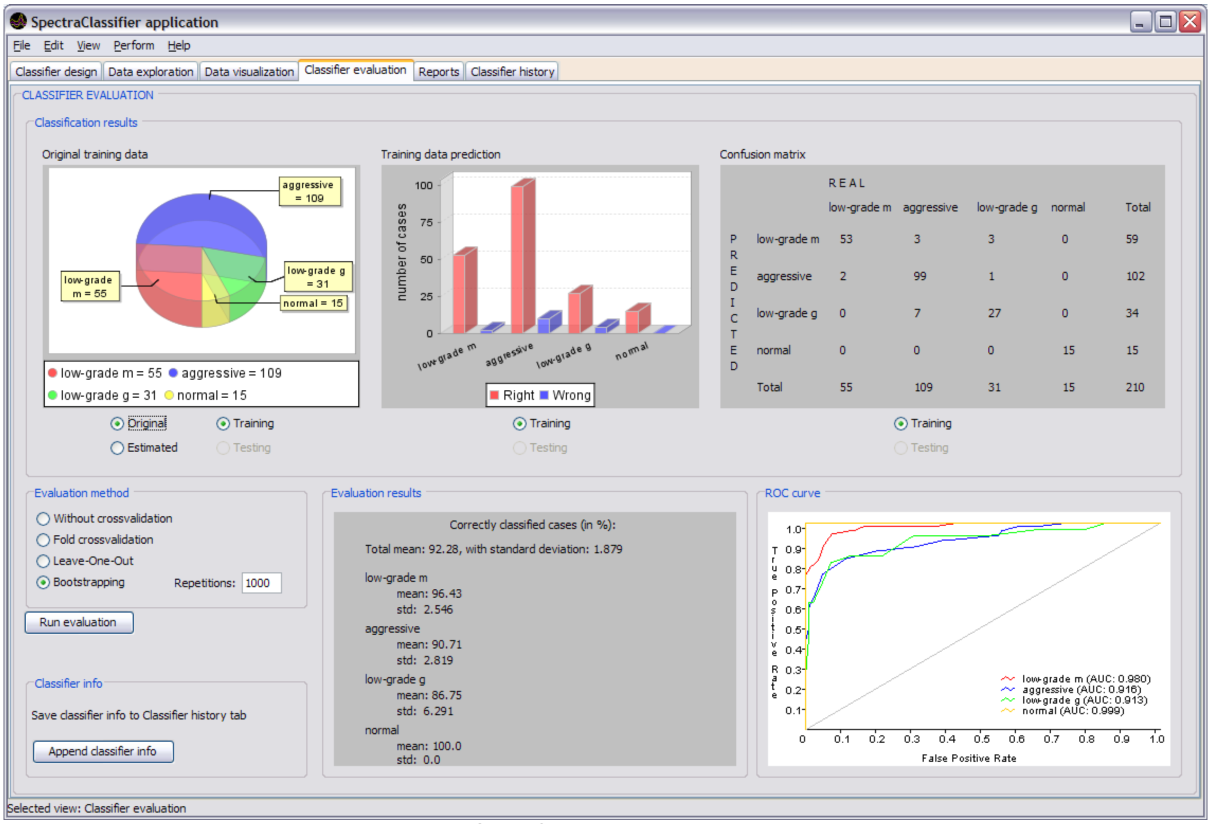

Classifier evaluation tab

6.2 Classification results

The following figure shows the number of cases by class. You can see the information related with the original training data set, original testing data set, estimated training data set and estimated testing data set. The **Testing** radio button will be enabled only if you loaded a testing data set.

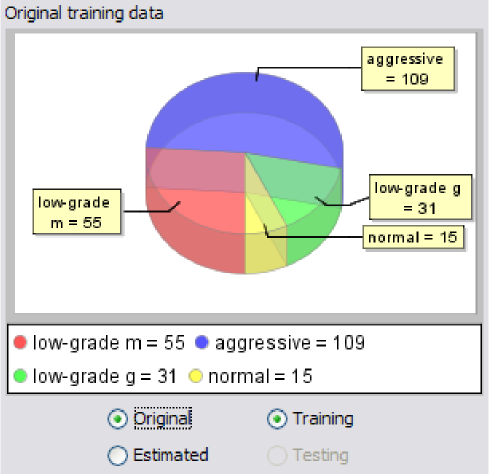

Count of cases per class
(from Classifier evaluation tab)

The subsequent figure allows to see the right and wrong number of cases predicted per class. The red colour indicates the correctly predicted ones and the blue colour indicates the wrongly ones. Depending on the radio button selection, you will see the prediction for the training or for the testing data set.

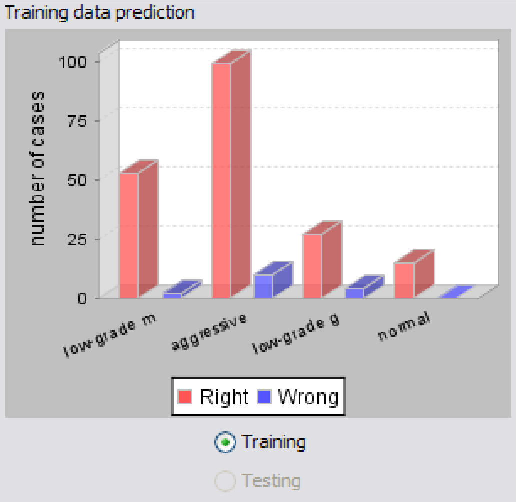

Data prediction per class
(from Classifier evaluation tab)

The next figure shows the **Confusion matrix**. Each row of the matrix represents the instances in a predicted class, while each column represents the real value of the instances in the original class. Confusion matrix allows seeing if the classifier is mislabelling cases.

© 2009 GABRMN - Universitat Autònoma de Barcelona

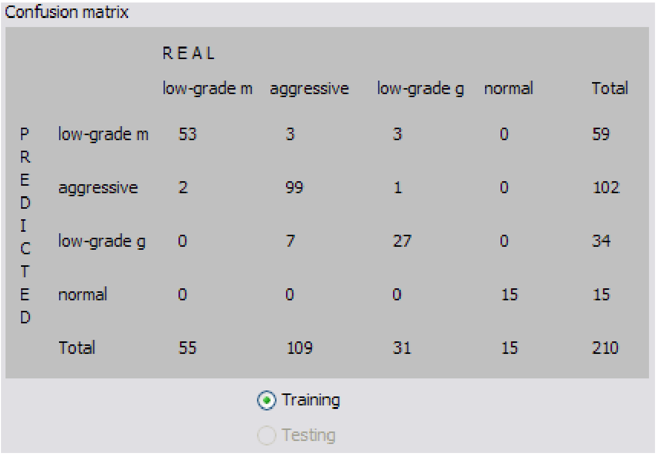

Confusion matrix

		REAL				
		low-grade m	aggressive	low-grade g	normal	Total
PREDICTED	low-grade m	53	3	3	0	59
	aggressive	2	99	1	0	102
	low-grade g	0	7	27	0	34
	normal	0	0	0	15	15
	Total	55	109	31	15	210

Confusion matrix (from Classifier evaluation tab)

6.3 Store classifiers info

The storage of multiple classifiers info can be useful to compare them. The practical way of do it with this applications is to add the info of a classifier already done to *Classifier history tab*, by means of *Append classifier info* button, in *Classifier evaluation tab*. See the following figure.

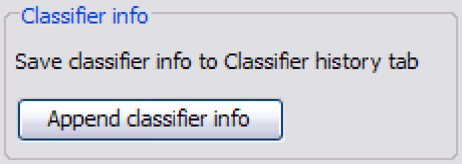

Append classifier info (from Classifier evaluation tab)

6.4 Evaluation method

You can evaluate the classifier using the following four methods: without cross validation (**Without crossvalidation** radio button), doing a cross validation with a specific number of repetitions (**Fold crossvalidation** radio button), using the maximum possible repetitions for the cross validation (**Leave-One-Out** radio button) or with a bootstrap method (**Bootstrapping** radio button).

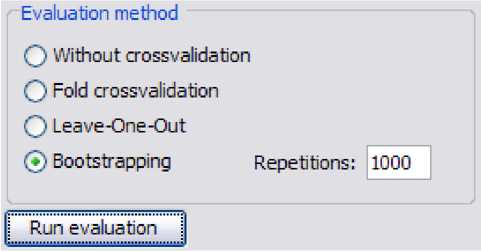

Evaluation method (from Classifier evaluation tab)

Cross-validation method is mainly used in settings where the goal is prediction, and one wants to estimate how accurately a predictive model will perform in practice. One round of cross-validation involves partitioning a dataset into complementary subsets, performing the analysis on one subset (training set), and validating the analysis on the other subset (testing set). In K-fold cross-validation, the original dataset is partitioned into K subsamples. Of the K subsamples, a single subsample is retained

as the testing data for testing the model, and the remaining K-1 subsamples are used as training data. The cross-validation process is then repeated K times (the folds), with each of the K subsamples used exactly once as the testing data. The K results from the folds then can be averaged to produce a single estimation [12].

Leave-One-Out (LOO) method is a special case of a K-fold cross-validation. It uses a single case from the original dataset as the testing data, and the remaining cases as the training data. This is repeated such that each case in the dataset is used once as the testing data. This is the same as a K-fold cross-validation with K being equal to the number of observations in the original dataset.

Bootstrapping: it is implemented by constructing a number N of bootstrap samples of the observed dataset (and of equal size to the observed dataset), each of which is obtained by random sampling with replacement from the original dataset (there is nearly always duplication of individual cases in a bootstrap dataset). The N results from the bootstrap samples then can be averaged to produce a single estimation [12]. Bootstrapping could be better at estimating error rates in a linear discriminant problem, outperforming cross-validation [13].

The results of the evaluation will be shown in the subsequent panel. See the following figure. For a four-class classifier (low grade meningioma, aggressive, low grade glioma and normal tissue) using 2 TE (Long plus Short), doing the evaluation with the Bootstrapping method, the overall mean accuracy for the correctly classified cases is 92.28%, with an standard deviation of 1.879%. By class, low grade meningioma was classified with a mean accuracy of 96.43% and a standard deviation of 2.546%.

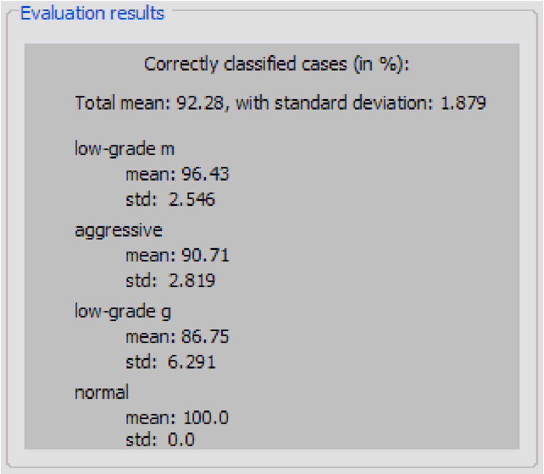

Evaluation results (from Classifier evaluation tab)

Note that the mean and the standard deviation will be displayed if the cross validation or the bootstrap has been performed. When no-crossvalidation has been performed you will only see the mean.

Detailed example of K-fold cross-validation and LOO

For the K-fold cross-validation, we will exemplify a 5-fold cross-validation. The dataset used in this example contains 217 cases. Let's divide the dataset in 5 subsamples (S1, S2, S3, S4, and S5), therefore K=5 (please see next figure). Of the 5 subsamples, a single subsample is retained as testing data for testing the model, and the remaining 4 subsamples are used as training data. The cross-validation process is then repeated 5 times (the folds), that we called stages in the next figure. At every stage, 43 (217/5) different cases are used to test the model developed with the 174 remaining cases, obtaining for each stage the mean of the cases correctly predicted of the test group. Then, the total mean and the standard deviation of the correctly predicted cases can be calculated.

© 2009 GABRMN - Universitat Autònoma de Barcelona

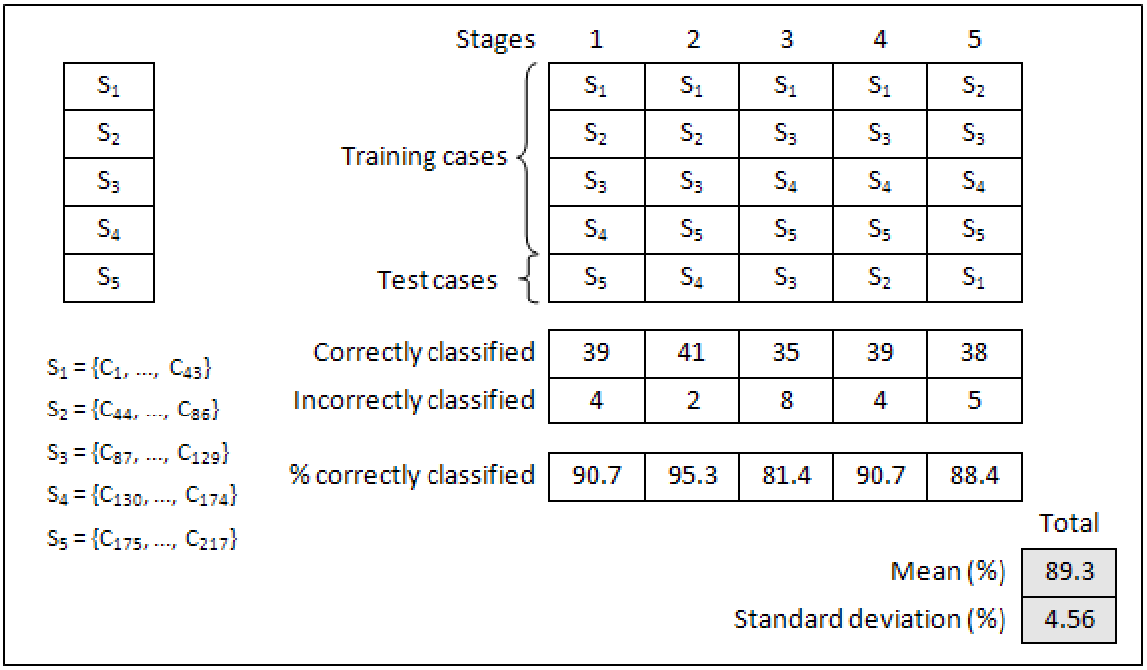

Example of a 5-fold cross-validation. (S: subset. C: case.)

In the LOO, the sample is partitioned in the number of cases in the original dataset (217 in this example), and a single case from the original dataset is used as testing data, while the remaining cases are used as training data (please see next figure). Then, the total mean and the standard deviation of the correctly predicted cases can be calculated as in the K-fold cross-validation method.

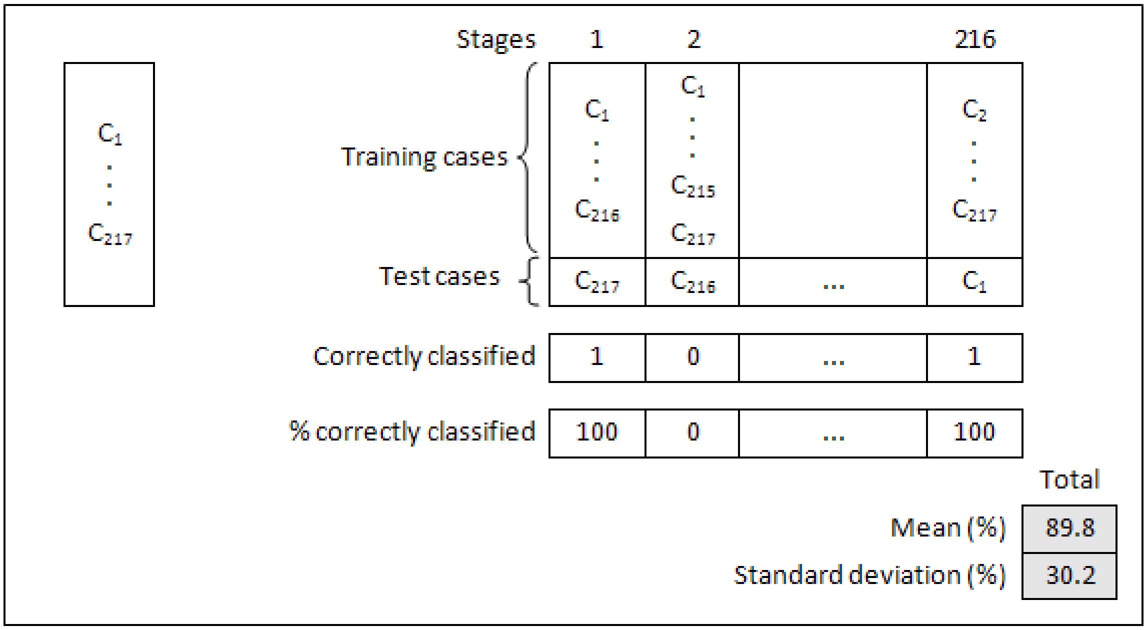

Example of Leave-One-Out. (C: case.)

© 2009 GABRMN - Universitat Autònoma de Barcelona

6.5 ROC curve

A receiver operating characteristic (ROC), or simply ROC curve, is a graphical plot of the sensitivity vs. (1 - specificity) for a binary classifier system as its discrimination threshold is varied. It is also known as a Relative Operating Characteristic curve, because it is a comparison of two operating characteristics (TPR = true positive rate & FPR = false positive rate) as the criterion changes.In the case of a classifier with more than two classes, each instance is analysed from the perspective of belong to a class or not while the threshold varies.

In this application, each ROC curve drawn per class shows the results of the probabilities generated by the classifier dichotomising each class versus the rest of classes. In the following image you can see three ROC curves, the red one corresponds to class mm (as shown in the legend), the blue one corresponds to class gl-me, etc.

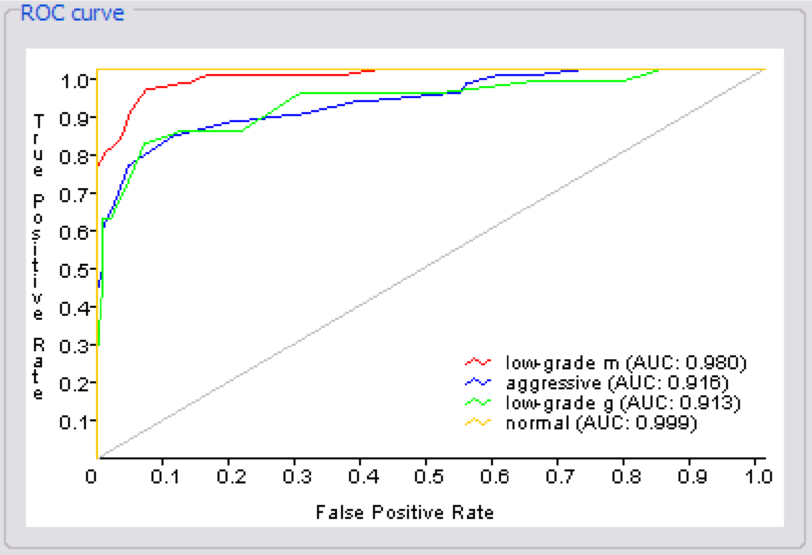

ROC curve (from Classifier evaluation tab)

TAB: Reports

Part VII

7 TAB: Reports

7.1 Overview

Reports tab is the fifth tab of the application. Its purpose is to allow the user to export the results of Fisher LDA and/or PCA, as shown in the following figure.

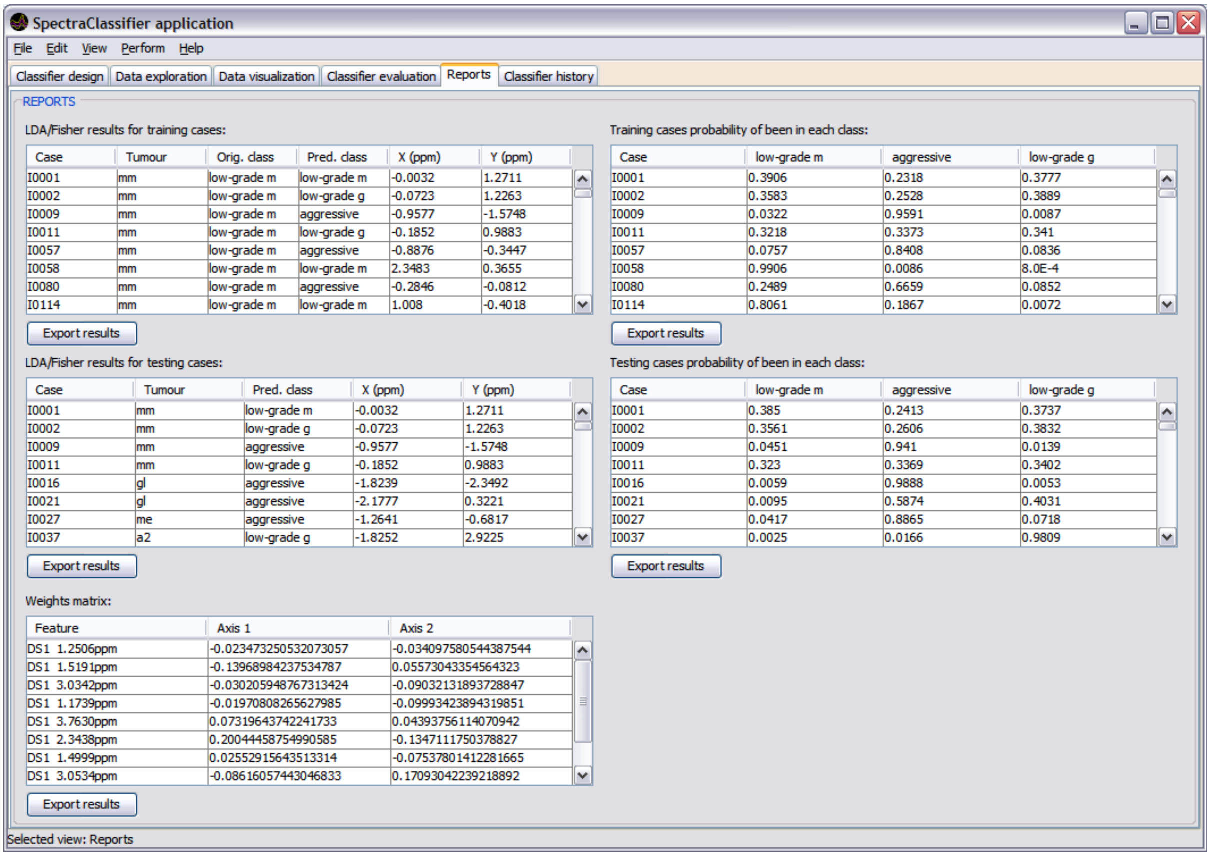

Reports tab

7.2 Fisher LDA results

The following report shows the classifier results for training and testing cases. Each row corresponds to a different case.

The **Case** column is the identifier of the case.
The **Tumour** column is the tumour type (if applicable, in case of test cases without tumour type, this column will be missing, this is the case of the example shown in the next figure).
The **Orig. class** column is the corresponding original class, depending of the tumour type.
The **Pred. class** column is the predicted class, obtained with Fisher LDA method.
The **X**, **Y** and **Z** columns correspond to the coordinates (in ppm). Their number will vary depending on the number of classes (It will be the number of classes - 1).

The **Export results** buttons can be used to export these data to a text file.

© 2009 GABRMN - Universitat Autònoma de Barcelona

LDA/Fisher results for training cases:

Case	Tumour	Orig. class	Pred. class	X (ppm)	Y (ppm)	Z (ppm)
I0001	mm	low-grade m	low-grade m	-1.8456	2.2477	2.7405
I0002	mm	low-grade m	low-grade m	-1.138	2.5896	4.6397
I0009	mm	low-grade m	aggressive	0.7445	0.0967	3.3835
I0011	mm	low-grade m	low-grade m	0.3692	1.3357	1.9433
I0057	mm	low-grade m	low-grade m	0.4584	1.349	2.6026
I0058	mm	low-grade m	low-grade m	2.035	2.8216	3.7323
I0080	mm	low-grade m	low-grade m	0.4609	0.558	3.2042
I0114	mm	low-grade m	low-grade m	-0.1572	1.1883	3.5254

Export results

LDA/Fisher results for testing cases:

Case	Tumour	Pred. class	X (ppm)	Y (ppm)	Z (ppm)
I0001	mm	low-grade m	-1.8456	2.2477	2.7405
I0002	mm	low-grade m	-1.138	2.5896	4.6397
I0009	mm	aggressive	0.7445	0.0967	3.3835
I0011	mm	low-grade m	0.3692	1.3357	1.9433
I0016	gl	aggressive	-0.2577	-1.4764	2.8769
I0021	gl	aggressive	-0.1958	-1.106	1.7432
I0027	me	aggressive	-1.9434	-0.7919	5.0593
I0037	a2	low-grade g	-1.503	0.3658	0.5243

Export results

LDA/Fisher results for training and testing cases (from Reports

7.3 Fisher LDA probabilities

The following report shows the probabilities of each case of being in each class (for training and testing cases). Each row corresponds to a different case.

The **Case** column is the identifier of the case.
The rest of columns correspond to the probabilities of being in each class. It will be a column per class.

The **Export results** buttons can be used to export these data to a text file.

Training cases probability of been in each class:

Case	low-grade m	aggressive	low-grade g
I0001	0.3906	0.2318	0.3777
I0002	0.3583	0.2528	0.3889
I0009	0.0322	0.9591	0.0087
I0011	0.3218	0.3373	0.341
I0057	0.0757	0.8408	0.0836
I0058	0.9906	0.0086	8.0E-4
I0080	0.2489	0.6659	0.0852
I0114	0.8061	0.1867	0.0072

Export results

Testing cases probability of been in each class:

Case	low-grade m	aggressive	low-grade g
I0001	0.385	0.2413	0.3737
I0002	0.3561	0.2606	0.3832
I0009	0.0451	0.941	0.0139
I0011	0.323	0.3369	0.3402
I0016	0.0059	0.9888	0.0053
I0021	0.0095	0.5874	0.4031
I0027	0.0417	0.8865	0.0718
I0037	0.0025	0.0166	0.9809

Export results

LDA/Fisher probabilities for training and testing cases (from Reports tab)

Determining probabilities

The general formulas used for determining these probabilities are:

Bayes' theorem:

$$P(G_j \mid X) = \frac{P(X \mid G_j)P(G_j)}{P(X)}$$

where:

$P(G_j)$ is the prior probability or marginal probability of G_j.

$P(G_j \mid X)$ is the conditional probability of G_j, given X. It is also called the posterior probability because it is derived from or depends upon the specified value of X.

$P(X \mid G_j)$ is the conditional probability of X given G_j.

$P(X)$ is the prior or marginal probability of X.

Intuitively, Bayes' theorem in this form describes the way in which one's beliefs about observing ' G_j' are updated by having observed 'X'.

In *SpectraClassifier*, the implemented method to obtain these probabilities does not take into account the prior probability. It is assumed that this prior probability is the same for each group.

$$P(G_j \mid X) = \frac{P(X \mid G_j)\cancel{P(G_j)}}{\sum_j P(X \mid G_j)\cancel{P(G_j)}}$$

For obtaining $P(X \mid G_j)$, the following formula was used:

$$P(X \mid G_j) = \frac{1}{\sigma\sqrt{(2\pi)^D}} \exp\left(\frac{1}{2\sigma^2}\left|X - \mu_j\right|^2\right)$$

Since Fisher LDA uses distances to the mean of the classes to predict which class a case belongs, it describes a radial basis function. For this reason the Mahalanobis distance was replaced by Euclidean distance in the normal distribution function to approximate $P(X \mid G_j)$.

The calculation of the standard deviation is described by the following formula:

$$\sigma = \sqrt{\frac{1}{N}\sum_{i=1}^{N}(x_i - \mu)^2}$$

7.4 Weights matrix

The following report shows the weights matrix, associated to the corresponding feature. Those features are expressed in ppm and the number of axes depends on the number of classes (It will be the number of classes - 1).

The Export Results button can be used to export this data to a text file.

© 2009 GABRMN - Universitat Autònoma de Barcelona

In case of a feature extraction with PCA the Feature column will be missing.

Weights matrix:

Feature	Axis 1	Axis 2
DS1 1.2506ppm	-0.023473250532073057	-0.034097580544387544
DS1 1.5191ppm	-0.13968984237534787	0.05573043354564323
DS1 3.0342ppm	-0.030205948767313424	-0.09032131893728847
DS1 1.1739ppm	-0.01970808265627985	-0.09993423894319851
DS1 3.7630ppm	0.07319643742241733	0.04393756114070942
DS1 2.3438ppm	0.20044458754990585	-0.1347111750378827
DS1 1.4999ppm	0.02552915643513314	-0.07537801412281665
DS1 3.0534ppm	-0.08616057443046833	0.17093042239218892

Export results

Weights matrix (from Reports tab)

7.5 PCA results

The following three reports show the PCA results. The following figure shows the first one with the principal component functions.

PCA functions:

Principal component functions
PC1: 0.087*1.3273ppm(DS2) +0.087*1.3082ppm(DS2)
PC2: -0.127*1.5767ppm(DS2) -0.127*1.5575ppm(DS2)
PC3: 0.122*0.6753ppm(DS2) +0.119*0.6561ppm(DS2)
PC4: 0.115*0.7328ppm(DS2) +0.115*0.6945ppm(DS2)
PC5: -0.139*1.9986ppm(DS2) -0.138*1.9794ppm(DS2)
PC6: 0.135*2.3246ppm(DS1) +0.128*2.3054ppm(DS1)
PC7: 0.189*0.6945ppm(DS1) +0.183*0.6561ppm(DS1)
PC8: -0.202*4.0506ppm(DS2) -0.199*4.0123ppm(DS2)

Export results

PCA functions (from Reports tab)

The following figure shows two other PCA reports with the results of the numerical value obtained after applying each principal component function to every training and testing case.

Principal components of training cases:

Case	F1	F2	F3	F4	F5	F6	F7	F8	F9	F10
I0001	0.76	-0.8	-0.49	-0.16	-2.05	1.28	0.09	-3.41	1.88	-6.27
I0002	1.25	-1.63	0.11	0.2	-1.55	1.66	0.51	-1.39	1.23	-1.0
I0009	3.71	-1.71	-0.26	-0.12	-1.71	0.63	1.55	-1.69	0.13	-1.02
I0011	1.14	-1.06	0.8	0.79	-1.94	1.04	0.75	-2.69	3.33	0.16
I0057	1.4	-2.38	0.32	0.41	-1.81	1.33	0.45	-1.95	2.22	-0.22
I0058	1.84	-1.73	0.25	0.4	-1.8	1.11	0.27	-1.39	1.98	0.9
I0080	2.25	-1.67	-0.04	0.29	-1.79	0.41	-0.01	-1.83	1.76	-2.06
I0114	2.34	-2.0	0.0	0.2	-2.0	2.37	0.01	-1.59	2.15	-0.72

Export results

Principal components of testing cases:

Case	F1	F2	F3	F4	F5	F6	F7	F8	F9	F10
I0001	0.76	-0.8	-0.49	-0.16	-2.05	1.28	0.09	-3.41	1.88	-6.27
I0002	1.25	-1.63	0.11	0.2	-1.55	1.66	0.51	-1.39	1.23	-1.0
I0009	3.71	-1.71	-0.26	-0.12	-1.71	0.63	1.55	-1.69	0.13	-1.02
I0011	1.14	-1.06	0.8	0.79	-1.94	1.04	0.75	-2.69	3.33	0.16
I0016	3.62	-1.67	0.14	0.33	-1.28	0.18	1.06	-3.47	0.61	0.1
I0021	2.25	-2.41	0.81	0.94	-1.69	0.13	1.78	-2.68	1.33	-1.78
I0027	3.96	-1.87	0.02	0.26	-1.94	0.87	0.98	-0.05	1.74	-0.08
I0037	0.89	-0.82	-0.12	-0.09	-2.52	0.04	0.21	-2.53	2.86	-0.14

Export results

PCA results for training and testing cases (from Reports tab)

The **Export results** buttons can be used to export these data to a text file.

© 2009 GABRMN - Universitat Autònoma de Barcelona

Part VIII

8 TAB: Classifier history

8.1 Overview

Classifier history tab is the sixth tab of the application. Its purpose is to have a place to compare classifiers made with different parameters. Every time you create a new classifier, the *Append classifier info* button in Classifier evaluation tab will allow you to add the principal information of this classifier to *Classifier history tab*.

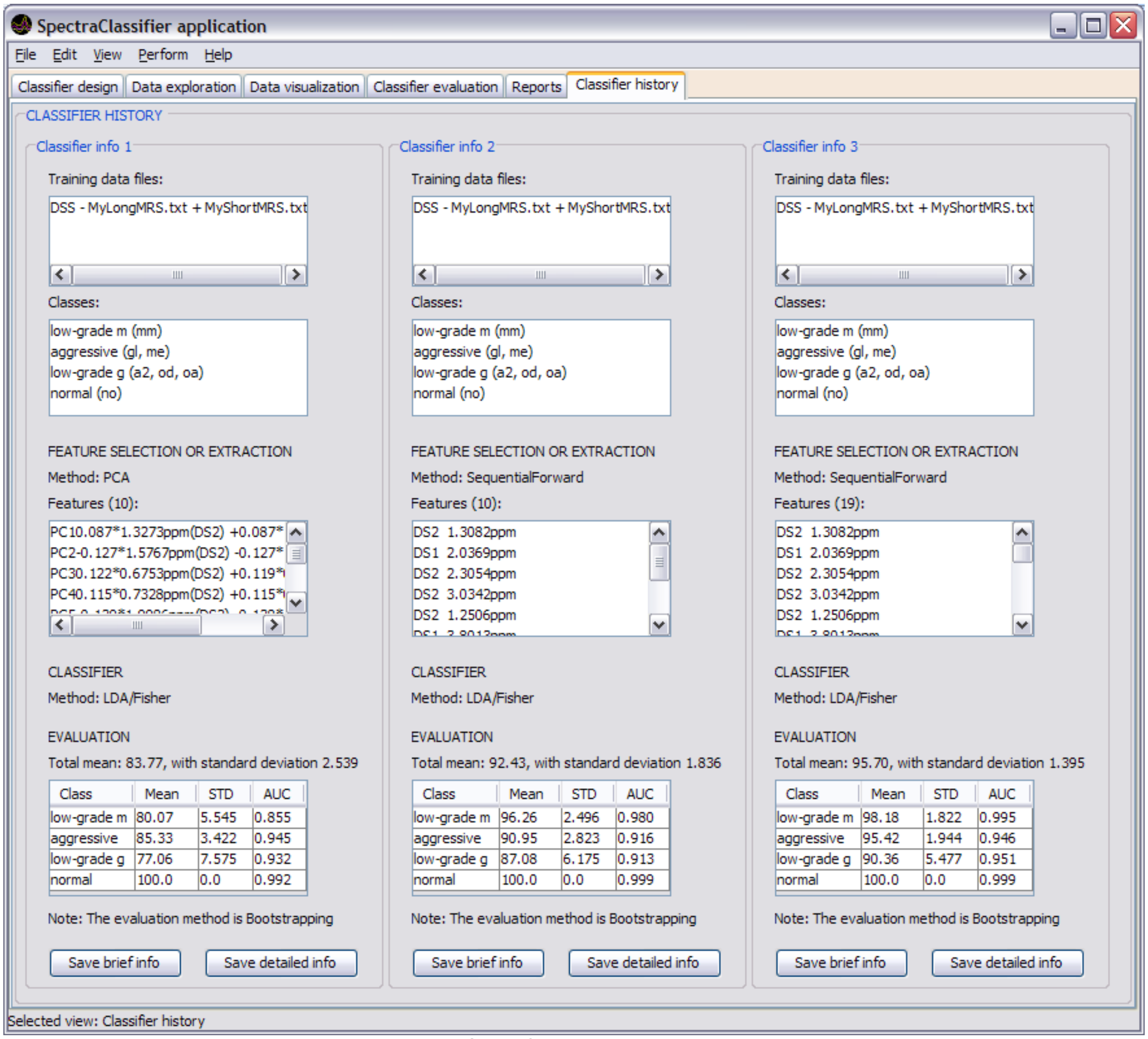

Classifier history tab

8.2 Classifier info

Every *Classifier info* is composed by the names of the training datasets used to create the classifier; the composition of the classes or groups; the features selection or extraction method, the number of features and the list of them; the classifier method; the results of the evaluation of the classifier with the Bootstrapping method (1000 repetitions), and the AUC (Area Under the Curve) from the ROC curve. See the following figure.

© 2009 GABRMN - Universitat Autònoma de Barcelona

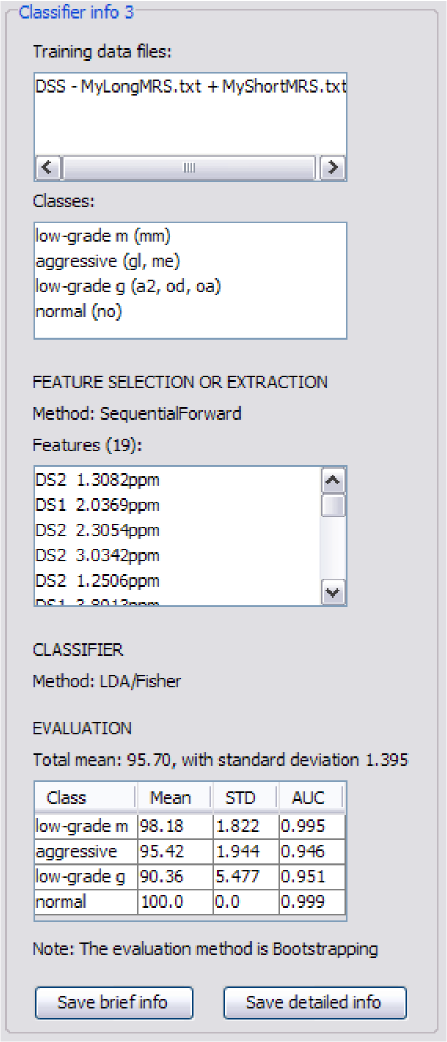

Classifier info
(from Classifier history tab)

The *Save brief info* button can be used to put this information into a text file. For instance, the corresponding text file for this classifier info will be as follows:

```
CLASSIFIER INFO:

Training data files:
DSS - MyLongMRS.txt + MyShortMRS.txt
null

Classes:
low-grade m (mm)
aggressive (gl, me)
low-grade g (a2, od, oa)
normal (no)

FEATURE SELECTION OR EXTRACTION
Method: SequentialForward
Number of features: 19
Features (in ppm): DS2 1.3082ppm, DS1 2.0369ppm, DS2 2.3054ppm, DS2 3.0342ppm,
```

© 2009 GABRMN - Universitat Autònoma de Barcelona

```
    DS2  1.2506ppm, DS1  3.8013ppm, DS2  3.5520ppm, DS1  1.5383ppm, DS1  1.9986ppm,
    DS1  1.2506ppm, DS1  2.4780ppm, DS2  3.8205ppm, DS1  3.0534ppm, DS1  2.3054ppm,
    DS2  2.4205ppm, DS1  2.1328ppm, DS1  2.9383ppm, DS2  0.9054ppm, DS2  3.6671ppm

CLASSIFIER
Method: LDA/Fisher

EVALUATION
Total mean: 95.70%, with standard deviation 1.395%

low-grade m
Mean: 98.18%
STD: 1.822%
AUC: 0.995

aggressive
Mean: 95.42%
STD: 1.944%
AUC: 0.946

low-grade g
Mean: 90.36%
STD: 5.477%
AUC: 0.951

normal
Mean: 100.0%
STD: 0.0%
AUC: 0.999

The evaluation method is Bootstrapping
```

The *Save detailed info* button saves the whole information into two XML files, one of them with the information of the classifier and the other with the information of the dataset used for training. The name of the dataset file will be the same that was typed for the classifier, plus "_dataset"; and the format of this file is explained in Format of the exported files section. The XML file with the classifier information has the following schema:

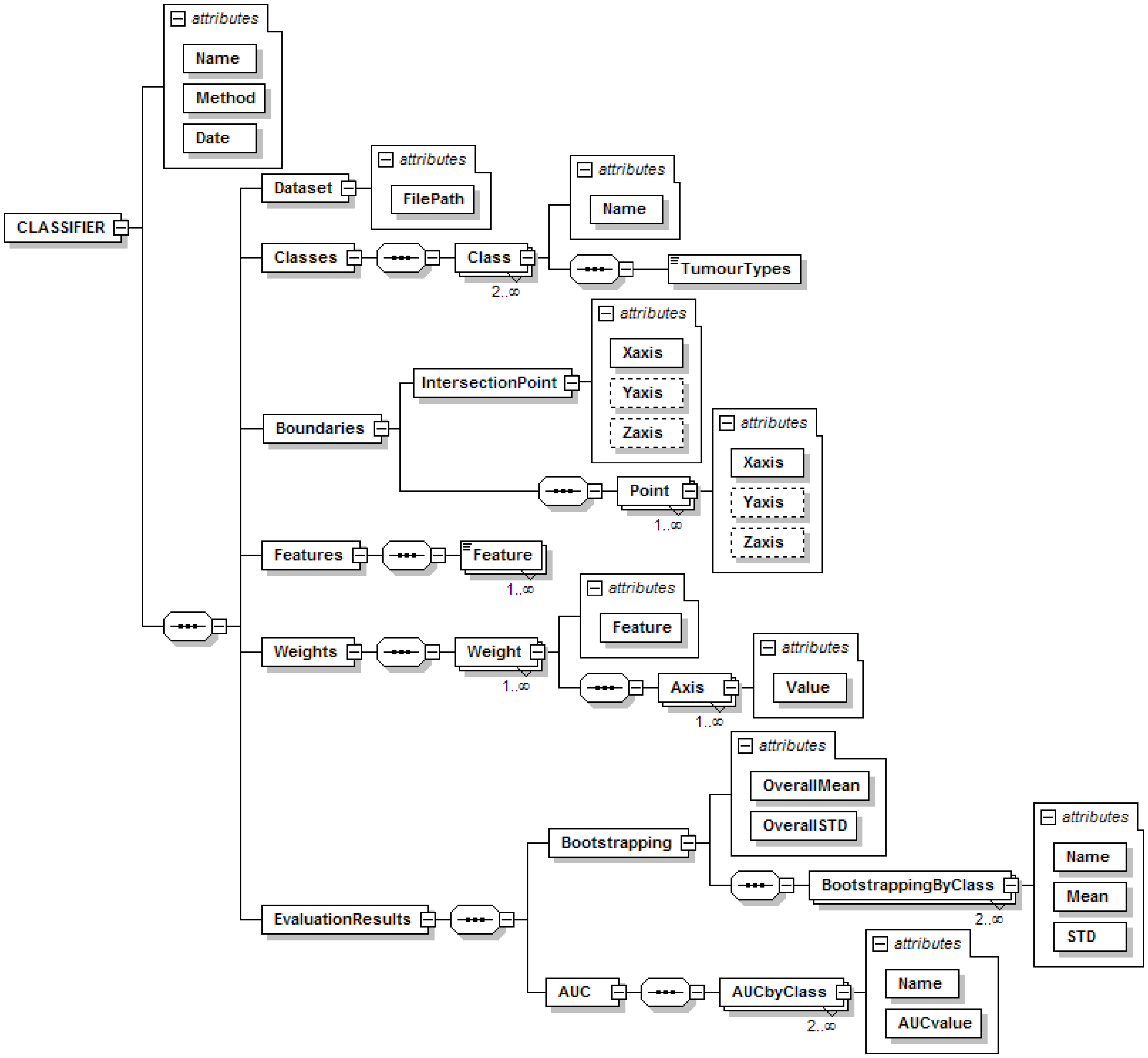

Schema of the classifier info

References

Part IX

9 References

[1] **Weka**

http://www.cs.waikato.ac.nz/ml/weka/

Weka is a collection of machine learning algorithms for data mining tasks. The algorithms can either be applied directly to a dataset or called from your own Java code. Weka contains tools for data pre-processing, classification, regression, clustering, association rules, and visualization. It is also well-suited for developing new machine learning schemes.

Weka is open source software issued under the GNU General Public License.

Ian H. Witten and Eibe Frank. **Data Mining: Practical machine learning tools and techniques.** 2nd Edition, Morgan Kaufmann, San Francisco, 2005.

[2, 3] **JavaStat and StatGraphics**

http://www2.thu.edu.tw/~wenwei/javastat/doc/

http://www2.thu.edu.tw/~wenwei/statgraphics/doc/

JavaStat and StatGraphics, implemented using Java, are open-source, platform-neutral libraries for performing basic statistics. Commonly used statistical methods and plots in a variety of areas are implemented in the libraries within a framework designed to be easy to use, extend, and integrate with other user-friendly software.

[4] **KiNG (Kinemage, Next Generation)**

http://kinemage.biochem.duke.edu/software/king.php

KiNG (Kinemage, Next Generation) is an interactive system for three-dimensional vector graphics. It supports a set of graphics primitives that make it suitable for many types of graphs, plots, and other illustrations; although its first use was to display macromolecular structures for biophysical research.

[5] Tate AR, Underwood J, Acosta DM, Julià-Sapé M, Majós C, Moreno-Torres A, Howe FA, van der Graaf M, Lefournier V, Murphy MM, Loosemore A, Ladroue C, Wesseling P, Luc Bosson J, Cabañas ME, Simonetti AW, Gajewicz W, Calvar J, Capdevila A, Wilkins PR, Bell BA, Rémy C, Heerschap A, Watson D, Griffiths JR, Arús C. **Development of a decision support system for diagnosis and grading of brain tumours using in vivo magnetic resonance single voxel spectra.** NMR Biomed. 2006 Jun;19(4):411-34.

[6] García-Gómez JM, Luts J, Julià-Sapé M, Krooshof P, Tortajada S, Robledo JV, Melssen W, Fuster-García E, Olier I, Postma G, Monleón D, Moreno-Torres A, Pujol J, Candiota AP, Martínez-Bisbal MC, Suykens J, Buydens L, Celda B, Van Huffel S, Arús C, Robles M. **Multiproject-multicenter evaluation of automatic brain tumor classification by magnetic resonance spectroscopy.** *Magnetic Resonance Materials in Physics, Biology and Medicine* (MAGMA). 2009 Feb;22(1):5-18. Epub 2008 Nov 7.

[7] Richard A. Johnson and Dean W. Wichern. **Applied Multivariate Statistical Analysis** (6th Edition). Prentice Hall, April 2007.

[8] van den Boogaart A, van Hecke P, van Huffel S, Graveron-Demilly D, van Ormondt D, de Beer R: **MRUI: a graphical user interface for accurate routine MRS data analysis**. *Magnetic Resonance Materials in Physics, Biology and Medicine* (MAGMA). 1996, **4**:318.

[9] Zhao Q, Patriotis P, Arias-Mendoza F, Stoyanova R, Brown TR: **3D Interactive Chemical Shift Imaging: A Comprehensive Software Program for Data Analysis and Quantification** In: *48th ENC Experimental Nuclear Magnetic Resonance Conference: 2007*; 2007.

© 2009 GABRMN - Universitat Autònoma de Barcelona

[10] Simões RV, Delgado-Goñi T, Lope-Piedrafita S, Arús C: **^{1}H-MRSI pattern perturbation in a mouse glioma: the effects of acute hyperglycemia and moderate hypothermia**. *NMR in Biomedicine* 2009:DOI: 10.1002/nbm.1421.

[11] **TopSpin**. In. Rheinstetten, Germany: Bruker BioSpin http://www.bruker-biospin.com.

[12] Duda, R.O., P.E. Hart, and D.G. Stork. **Pattern Classification** (Second edition). New York: John Wiley & Sons, 2001.

[13] Efron, B. and R.J. Tibshirani, **An introduction to the bootstrap**. Monographs on statistics and applied probability, 57. Chapman & Hall,1998.
